# High-coverage ancient genomes reveal divergent population histories and prehistoric starch-related genetic variation in Japan

**DOI:** 10.1073/pnas.2606162123

**Published:** 2026-07-23

**Authors:** Koji Ishiya, Fuzuki Mizuno, Jun Gojobori, Masahiko Kumagai, Yasuhiro Taniguchi, Osamu Kondo, Masami Matsushita, Takayuki Matsushita, Li Wang, Kunihiko Kurosaki, Shintaroh Ueda

**Affiliations:** ^a^ Sapiens Life Sciences, Evolution and Medicine Research Center, Kanazawa University, Kanazawa 920-8640, Japan; ^b^ Institute for the Study of Ancient Civilizations and Cultural Resources, Kanazawa University, Kanazawa 920-1192, Japan; ^c^ Department of Biological Sciences, Graduate School of Science, The University of Tokyo, Bunkyo-ku 113-8654, Japan; ^d^ Department of Legal Medicine, Toho University School of Medicine, Ōta-ku 143-0015, Japan; ^e^ Research Center for Integrative Evolutionary Science, The Graduate University for Advanced Studies (SOKENDAI), Hayama 240-0193, Japan; ^f^ Research Center for Advanced Analysis, National Agriculture and Food Research Organization, Tsukuba 305-8517, Japan; ^g^ Department of Archaeology, Faculty of Letters, Kokugakuin University, Shibuya-ku 150-8440, Japan; ^h^The Doigahama Site Anthropological Museum, Shimonoseki 759-6121, Japan; ^i^ Department of Evolutionary Studies of Biosystems, The Graduate University for Advanced Studies (SOKENDAI), Hayama 240-0193, Japan

**Keywords:** ancient DNA, high-coverage ancient genome, Jomon and Yayoi ancestry, dietary adaptation, Japanese Archipelago

## Abstract

Prehistoric population movements played a central role in shaping the genetic diversity across Eurasia. As the eastern terminus of the Eurasian continent, the Japanese archipelago provides a pivotal context for reconstructing the genetic interplay between prehistoric indigenous lineages and continental migrations. However, high-resolution genomic evidence from mainland Japan is limited due to poor DNA preservation. In the present study, we report two low-contamination, high-coverage ancient genomes from mainland Japan, namely an Initial Jomon individual (>67-fold) and a Middle Yayoi individual (>46-fold). The genomes enabled diploid genotyping and individual-level demographic inferences at unparalleled resolutions. Demographic reconstructions revealed that while both lineages underwent population contractions during the Last Glacial Maximum (LGM), their subsequent trajectories diverged; the Jomon lineage was characterized by long-term postglacial stability with no discernible expansion, whereas the Yayoi-related ancestral population exhibited gradual and sustained growth. Our analysis further identified a distinct genetic continuity between the Middle Yayoi and present-day mainland Japanese, refining the evolutionary trajectory of modern genetic profiles. Furthermore, the genomic profile of the Yayoi individual reflected a complex ancestral background shaped by continental interactions before their arrival on the archipelago. Notably, the higher salivary amylase (*AMY1*) copy number observed in the Initial Jomon individual suggests that *AMY1* copy number variation, potentially relevant to starch-rich diets, was already present before the large-scale expansion of rice farming in the Japanese Archipelago. Collectively, the findings demonstrate that high-coverage ancient genomic approaches can provide a powerful framework for deciphering the contrasting population histories and evolutionary trajectories of human lifestyles.

## Significance

How prehistoric migrations and dietary shifts shaped genetic diversity is a central question in human history. However, the lack of high-coverage ancient genomes from East Eurasia limits high-resolution reconstruction. This study presented high-coverage genomes from Jomon and Yayoi individuals, revealing contrasting population histories that align with the distinct cultural backgrounds of indigenous lineages and continental ancestry. Notably, the higher *AMY1* copy number observed in the Initial Jomon individual suggests that copy number variation at this salivary amylase locus, potentially relevant to starch-rich diets, was already present before rice farming became widely established in the Japanese Archipelago. By providing high-coverage ancient genomic evidence, this study elucidated how the interplay of cultural shifts and migrations formed the basis of modern Japanese populations.

The prehistoric peopling of East Eurasia remains one of the most complex chapters in human evolutionary history (1, 2). Although the broad dispersal of modern humans into Eurasia approximately 60,000 years ago is well established, recent paleogenomics research has clarified the complex population dynamics and genetic transitions across East Eurasia since the Late Pleistocene (3–8). The studies have revealed the history of large-scale population shifts and admixtures that shaped the genomic landscape of the continent. In this context, the Japanese archipelago, situated at the eastern terminus of these routes, represents a pivotal geographic location for understanding how deep indigenous lineages interacted with later continental migration. Archaeological records have identified two transformative periods that define the character of the region, namely the Jomon (c. 16,000 to 3,000 years BP) and the subsequent Yayoi (c. 3,000 to 1,700 years BP) (9–12). The transition from the Paleolithic to the Jomon period was marked by the appearance of pottery (13, 14), leading to a long-standing hunter-gatherer culture noted for its early sedentary lifestyle and specialized resource utilization. This was later followed by the Yayoi period, characterized by the introduction of paddy rice cultivation in the Eurasian continent. The transitions are thought to represent a fundamental biological and demographic restructuring, potentially providing the genomic foundation for modern populations in the region.

Despite the significance of the Japanese mainland in these events, reconstructing its genomic history at high resolution has been limited by poor DNA preservation. The warm, humid, and acidic soil characteristics of the mainland are notoriously detrimental to the survival of ancient DNA (15), often limiting the available information to low-coverage genome-wide data or mitochondrial DNA sequences (16–18). Although a high-coverage ancient genome has been recovered from the cooler climate of Hokkaido (19), the mainland, the primary theater for the Jomon–Yayoi transition, lacks the high-depth resources necessary for sophisticated genomic analyses. Low-coverage data, though useful for broad ancestry mapping, preclude precise diploid genotyping, copy number variation (CNV) analysis, and individual-level demographic modeling. Such high-resolution analyses are essential for elucidating how cultural and genetic exchanges shaped the interplay between genomic variation and population history in East Eurasia.

In the present study, we addressed this critical gap by presenting two high-coverage ancient genomes from mainland Japan, namely an Initial Jomon individual (>67-fold) and a Middle Yayoi individual (>46-fold). The data represent an unparalleled genomic resource for East Eurasia, enabling the transition from simple admixture models to high-resolution evolutionary inferences. By leveraging the depth of these genomes, we clarified the genetic divergence between indigenous lineages and continental ancestry, reconstructed post-Last Glacial Maximum (LGM) demographic trajectories, and revealed higher *AMY1* copy numbers. Our findings demonstrated that high-coverage ancient genomic approaches can uncover the intricate relationships across cultural shifts, environmental changes, and genomic formation of modern populations.

## Results

### High-Coverage Ancient Genomes From Mainland Japan.

**Fig. 1.**
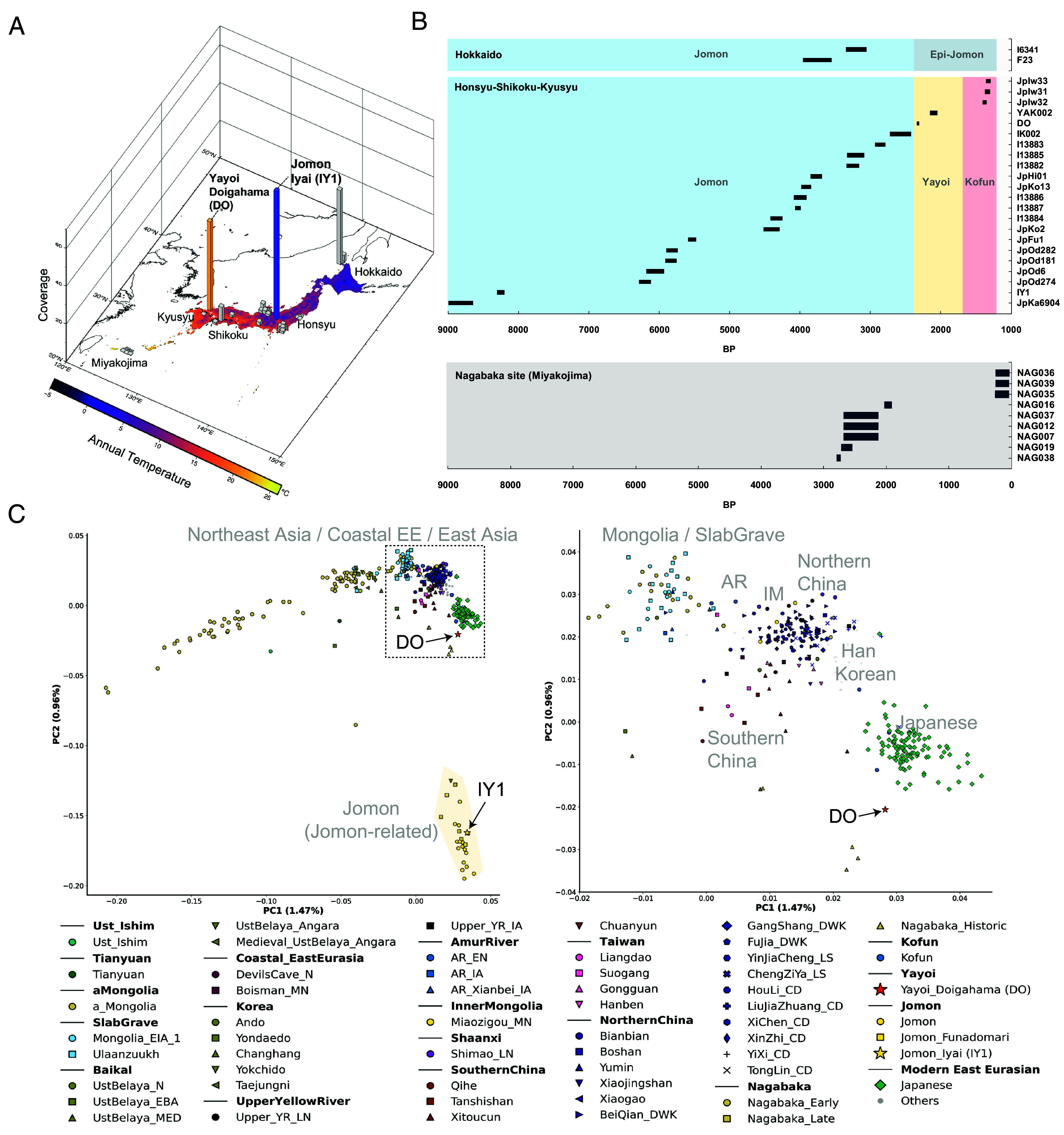
Spatiotemporal distribution and ancestral contexts of ancient genomes in Japan. (*A*) Archeological site locations and autosomal coverage (3D bars). Heatmap indicates 1991–2020 mean annual temperatures (yellow: warm; blue: cool; Japan Meteorological Agency). (*B*) Temporal and regional distribution of representative ancient human samples in Japan. Hokkaido and Miyakojima are plotted separately due to distinct cultural sequences. (*C*) PCA of East Eurasian populations. Ancient individuals are projected onto the space defined by present-day samples. Present-day Japanese individuals are shown as green diamonds, whereas other present-day populations are shown as gray dots. Newly sequenced IY1 and DO are highlighted as stars and arrows. The yellow-shaded region indicates the cluster of Jomon and Jomon-related individuals in the *Left* panel. The *Right* panel magnifies the dashed region from the *Left* panel, focusing on Northeast and East Asian relationships.

We performed high-coverage genome sequencing of specimens from the initial Jomon period (IY1) and the Middle Yayoi period (DO) from central and southwestern mainland Japan (Fig. 1*A*). IY1 is one of the oldest buried human remains from the Initial Jomon period (Fig. 1*A*). The Doigahama site, from which the DO was excavated, is also known as a site for migratory Yayoi people, and the individual could be a representative genomic resource for the migratory Yayoi people (Figs. 1 *A* and *B*). Genomic DNA was extracted from the petrous bones of both samples and shotgun-sequenced genomes showed high coverage (IY1: autosome average 67.94-fold; DO: autosome average 46.82-fold) (Table 1, *SI Appendix*, Figs. S1–S3 and Datasets S1 and S2). The authenticity of the ancient DNA was validated by confirming that the sequencing reads exhibited postmortem deamination patterns (*SI Appendix*, Fig. S4). Furthermore, both individuals exhibited less than 1% exogenous DNA contamination (IY1:0.727%; DO: 0.234% in the mitochondrial genome; male individual DO: 0.420% in the X chromosome) (Table 1). The maternal mitochondrial haplogroups of IY1 and DO were N9b and D4b2b1, respectively (Table 1). The results of the mitochondrial haplogroup assignments were consistent with those of previous studies on mitochondrial genomes (17, 18). We assigned the Y chromosome haplogroup of the male individual DO to the D1a2a lineage observed in modern Japanese individuals (*SI Appendix*, Figs. S5 and S6).

**Table 1.** Summary of sequenced samples in the present study

Sample ID			IY1	DO
Era			Initial Jomon	Middle Yayoi
Date (cal. yr B.P.)			8,300-8,200	2,306-2,238
Sex (Ry)			Female (0.002)	Male (0.090)
Contamination (%)		mtDNA	0.73	0.23
		chrX	N.A.	0.42
	mtDNA		1833.73	2735.05
	autosome		67.94	46.82
	rsid SNPs (dbSNP ver. 138)		3,058,278	2,985,236
Coverage	% of covered genome (w/o N sites)	0X	2.67	2.70
		>1X	97.33	97.30
		>5X	95.93	95.31
		>15X	92.88	91.04
		>30X	87.59	85.10

### Ancestral Contexts of Jomon and Yayoi Individuals.

Principal component analysis (PCA) using East Eurasian populations (Fig. 1*C* and Dataset S3) clearly distinguished the Jomon from other Northeast and East Asian populations. Notably, the Jomon cluster included an ancient Yokchido individual from the Korean Peninsula, suggesting prehistoric genetic interactions or migration across the sea. In contrast, the Yayoi individual (DO) plotted closer to continental East Asian populations than to the Jomon populations, reflecting a distinct genetic shift associated with the Yayoi period.

**Fig. 2.**
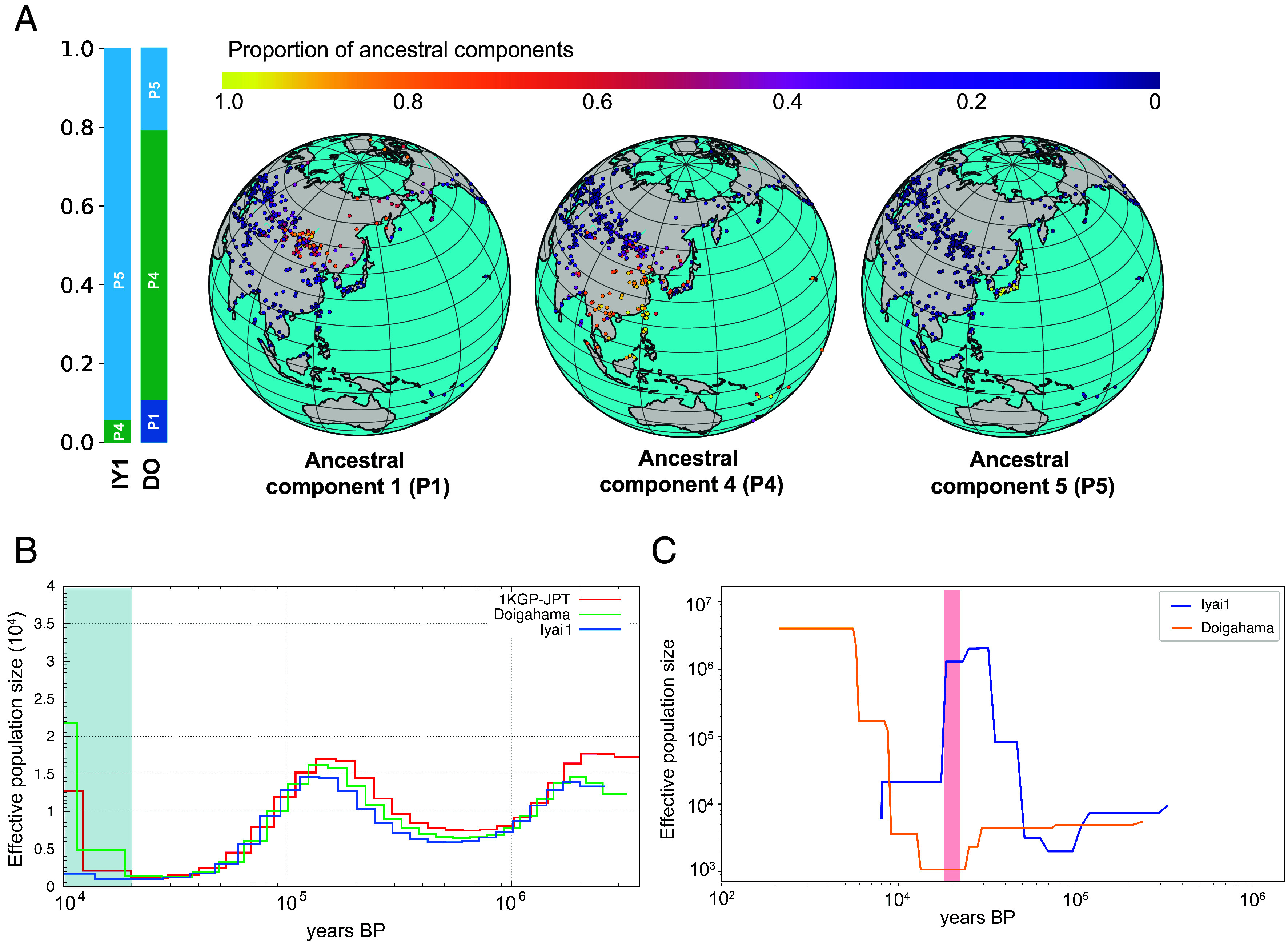
Ancestral compositions and demographic reconstructions. (*A*) Bar plots and distribution of ADMIXTURE ancestral proportions (*K* = 6) for IY1 (Jomon) and DO (Yayoi). Components P1, P4, and P5 are detailed in *SI Appendix*, Fig. S7. (*B*) Long-term population size histories inferred by PSMC. Lines: present-day Japanese (red: NA18939), Yayoi (green: DO), and Jomon (blue: IY1). (*C*) Recent demographic trajectories spanning the LGM inferred by SMC++. Blue: IY1; orange: DO. Shaded areas in (*B* and *C*) represent the period around the LGM.

Admixture analysis (Fig. 2*A* and *SI Appendix*, Figs. S7–S9) revealed that while both the IY1 and DO shared genetic components found in modern Japanese, they exhibited distinct ancestral profiles. A specific component (P1) prevalent in Neolithic-related Ancient Northeast Asian (ANEA) populations from the Baikal and Mongolian (*SI Appendix*, Fig. S10) regions was identified in DO but remained absent in the Jomon. This suggested that ANEA-related lineages likely represent a contributing source to the genetic formation of Yayoi, distinguishing their ancestry from that of the indigenous Jomon.

Furthermore, a shared component (P4), identified exclusively in high-coverage Jomon and Yayoi genomes but undetected in low-coverage samples (*SI Appendix*, Fig. S7), was prevalent across East Asia, Southeast Asia, and Oceania (Fig. 2*A* and *SI Appendix*, Fig. S9). This distribution suggested that the Jomon and Yayoi lineages retained common ancestral resources throughout the regions. Possible admixture modeling indicated that early diverging lineages (e.g., E2 and E21) serve as ancestral sources for the Jomon and Yayoi populations (*SI Appendix*, Figs. S11 and S12). Finally, the P5 component, which predominated in the Jomon, was also identified in the Yokchido individual from the Korean Peninsula, reflecting a Jomon-associated ancestry outside the Japanese archipelago (Fig. 2*A* and *SI Appendix*, Fig. S9).

Outgroup-*f3* and *f4* statistics revealed that while genetic affinities with East Eurasian populations remained stable throughout the Jomon period, it shifted remarkably during the Yayoi transition (*SI Appendix*, Figs. S13–S17 and Datasets S4–S7). Consistent with the Admixture results, while IY1 exhibited a high genetic affinity with other Jomon individuals, it lacked significant continental links, with the sole exception of the Yokchido individual from the Korean Peninsula (*SI Appendix*, Fig. S16 and Dataset S6). In contrast, the *f4* statistics for DO exhibited significant allele sharing with a broad range of Middle Neolithic populations from Northeast Asia and the Shandong Peninsula, including Miaozigou_MN and Xiaojingshan (*SI Appendix*, Fig. S16 and Dataset S6). This indicated a substantially increased genetic affinity with continental lineages compared to that in the Jomon period. In present-day Northeast Asia, the Daur people share the highest affinity with DO, further supporting a continental origin for the Yayoi ancestry (*SI Appendix*, Fig. S16 and Dataset S6). While Kofun-period samples showed distinct association patterns (*SI Appendix*, Fig. S17), outgroup-*f3* statistics revealed that DO retained a higher shared drift with Jomon and coastal East Eurasian lineages (e.g., Devil’s Gate and Boisman) than Kofun individuals (*SI Appendix*, Fig. S14 and Datasets S4 and S5).

### Contrasting Demographic Trajectories Post LGM.

The effective population sizes of the Jomon and Yayoi lineages showed no discernible difference until approximately 100 thousand years ago (kya) (Fig. 2*B*). However, their demographic trajectories diverged substantially after the LGM. Estimates for Jomon individuals revealed a lack of population growth and a declining trend during the LGM period (Figs. 2 *B* and *C*). In contrast, the Yayoi ancestral population exhibited a gradual and sustained increase in size after the LGM (Figs. 2 *B* and *C*). The pairwise sequentially Markovian coalescent (PSMC) demographic trajectories remained consistent across bootstrap replicates and first-window validations during the post-20 kya (*SI Appendix*, Figs. S18 and S19), confirming that the observed trends were not artifacts of demographic estimation. Our high-coverage ancient genomes revealed the contrasting demographic shifts between the two ancestral groups, reflecting their distinct lifestyles.

### Genetic Contribution of Prehistoric Peopling in the Japanese Archipelago.

Maximum likelihood phylogenetic analysis of East Eurasian populations suggested that the Jomon represents an early divergent lineage within East Asians (*SI Appendix*, Fig. S20). Moreover, the model inferred the migration edge from Jomon to modern Japanese, supporting a significant genetic contribution of the Jomon lineage to present-day populations. To estimate the timing of admixture events targeting present-day Japanese people, we analyzed the decay in weighted linkage disequilibrium (LD) using IY1, DO, and Han as ancestral proxies (*SI Appendix*, Fig. S21). The LD decay between IY1 and DO was 72.34 ± 6.82 generations, spanning the Yayoi and subsequent periods.

Furthermore, we performed two-way admixture modeling (qpAdm) for present-day Japanese, Yayoi, and Kofun individuals using Jomon, and published ancient East Eurasians as ancestral proxies (Dataset S8). For present-day Japanese, the two-way model was supported with West Liao River basin, Bronze Age (WLR_BA) as a plausible continental source, with admixture coefficients falling within the feasible range [0, 1]. In the Yayoi individual (DO), ancestral proxies from Northeast Asia, such as Amur River, Early Neolithic (AR_EN) and Haminmangha, Middle Neolithic (HMMH_MN), provided supported fits. Although candidates from the West Liao River (WLR_BA and WLR_MN) exhibited high tail probabilities, the nested models could not be rejected. Similarly, for Kofun-period individuals, the model identified WLR_BA and WLR_MN as supported continental sources, whereas other candidates (e.g., AR_IA and Miaozigou_MN) showed statistically plausible patterns, though ultimately less parsimonious. The results provided insights into the shifting landscape of continental genetic contributions that shaped Yayoi and post-Yayoi populations in the Japanese archipelago.

### *AMY1* Copy Numbers in Jomon and Yayoi Genomes.

**Fig. 3.**
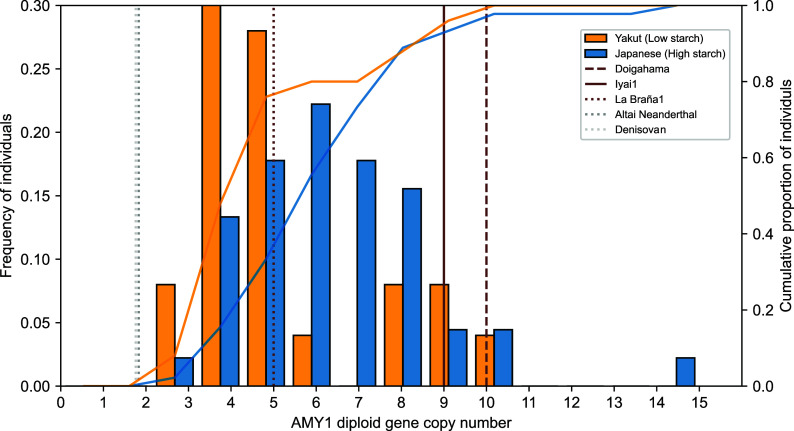
Comparison of *AMY1* copy number variations. Cumulative distributions of *AMY1* copy number polymorphisms in present-day East Asians (Yakut and Japanese). Vertical lines indicate estimated copy numbers for newly sequenced individuals (IY1 and DO), an ancient European hunter-gatherer (La Braña 1), and archaic hominins (Altai Neanderthal and Denisovan).

Taking advantage of the high-depth coverage of the IY1 and DO genomes, we inferred their copy numbers at the structurally complex, starch-digestion-related *AMY1* locus. Both individuals exhibited higher *AMY1* copy numbers compared to various present-day East Asian populations, across both high- and low-starch dietary groups, with IY1 carrying approximately nine copies and DO carrying approximately ten (Fig. 3 and Dataset S9). The values were notably higher than the approximately five *AMY1* copies identified in La Braña 1, a Mesolithic European hunter-gatherer (20). Although recent large-scale studies have suggested that the major expansion of *AMY1* copy numbers occurred globally after the onset of agriculture (21, 22), the analyses were geographically limited and largely lacked high-coverage ancient genomes from East Eurasia, particularly the Japanese archipelago. Our findings suggested that higher *AMY1* copy numbers, potentially relevant to the dietary use of starch-rich plant resources, were already present prior to the dispersal of rice farming in the Japanese Archipelago. Subsequently, the transition to agriculture may have further shaped these copy number variations in the Japanese archipelago.

## Discussion

Our high-coverage ancient genomes provided a milestone for understanding the contrasting demographic histories of the Jomon and Yayoi people (Figs. 1 and 2). Following the LGM, these lineages exhibited diverging trajectories. While the Initial Jomon ancestral population showed no remarkable numerical expansion, the Yayoi ancestral population experienced a gradual growth trend (Figs. 2 *B* and *C*). The patterns likely reflected how distinct ancestral lifestyles and environmental responses have shaped demographic history. Within the subsequent Jomon period, the lack of abrupt shifts in runs of homozygosity (ROH) levels among individuals from mainland Japan and Shikoku suggested that these populations avoided rapid demographic transitions or significant external influences from continental Eurasia, maintaining a relatively stable population size throughout the era (*SI Appendix*, Fig. S22). The results underscored a unique model of long-term population stability in the Japanese archipelago, which is distinct from the rapid expansion observed in postagricultural continental populations.

While previous research on *AMY1* evolution had predominantly focused on West Eurasian populations (21, 22), recent studies have suggested that *AMY1* copy number variation has more complex evolutionary histories, and that its relationship with agriculture and starch-rich diets may vary across populations and demographic backgrounds (22–24). In this study, our high-coverage genomes provide an East Eurasian perspective on ancient *AMY1* copy number variation. The findings suggested that, instead of a uniform response to agriculture, *AMY1* copy number variation may have followed regionally variable trajectories in the Japanese archipelago. Our results showed that the Initial Jomon individual (IY1) already possessed a higher *AMY1* copy count (approximately 9), which may be relevant to the dietary use of starch-rich plant resources in their lifestyles (Fig. 3 and Dataset S9). This genetic profile aligned with the archeological record at the Iyai rock shelter site, which yielded abundant plant remains, such as walnuts, chestnuts, or oaks. Furthermore, a recent report on the domestication of adzuki beans during the Jomon period (25) reinforced the significance of plant resources in their lifestyles. Our results suggested that regional dietary specialization including indigenous plant resources may have been associated with *AMY1* copy number variation related to starch digestion, independently of agricultural transitions such as those observed in West Eurasia. The subsequent transition to intensive rice farming in the Yayoi period may have further influenced *AMY1* copy number variation, as reflected by the higher copy number observed in DO (approximately 10). The results highlighted the possibility that different environmental and cultural contexts across Eurasia contributed to convergent yet temporally distinct diet-related genetic variation in humans.

Further, our high-coverage ancient genomes facilitated the precise reconstruction of the admixture events that shaped the Japanese population. We estimated that the Jomon–Yayoi admixture occurred approximately 65.52 to 79.16 generations ago while the divergence between present-day Han Chinese and Yayoi-related ancestors dated to 67.55 to 98.51 generations (*SI Appendix*, Fig. S21). These chronological intervals suggested that the formation of the Japanese population was likely a multistage process, whereby the ancestral continental pool potentially underwent its own genetic interactions before subsequent admixture within the archipelago. Our analysis identified a distinct Northeast Asian ancestral component in the Yayoi individual (DO) that was entirely absent in the Jomon representative (Fig. 2*A*). The prevalence of this component in ancient Northeast Asians, including individuals from the Ust’-Belaya site (26) and Mongolia (6), indicated that the lineage, or its related proxies, was broadly distributed across Northeast Eurasia before its integration into the Yayoi genomic profile. Geographically, the continental legacy within the Yayoi genome encompasses both northeast and coastal east Eurasia. The observed *f4* patterns reinforced the Yayoi individual’s close genetic affinity with Middle Neolithic groups from Inner Mongolia (e.g., Miaozigou_MN and HMMH_MN) and Early-to-Late Neolithic populations from Shandong, China (3, 4) (*SI Appendix*, Fig. S16). Among the present-day populations, the Daur people exhibit the strongest affinity with DO, suggesting a link with groups spanning from the Yellow River basin to the Mongolian plateau. Furthermore, outgroup-*f3* statistics linked the Yayoi individual to coastal East Eurasians, such as those from Devil’s Gate (27) and Boisman (6), more strongly than to the Kofun-period individuals (*SI Appendix*, Fig. S14). This connection supported the significant contribution of coastal and Mongolian Neolithic ancestry to the Yayoi people. The impact of these continental migrations exhibited a clear regional contrast within the Japanese archipelago. Our population modeling supported the idea that the mainland Yayoi and Kofun populations show genetic influences from the Northeast Asian and Inner Mongolian groups dating from the Neolithic age (Dataset S8). In contrast, the BeiQian group, a proposed source for the insular Nagabaka population (8), showed no clear genetic relationship with the mainland Yayoi and Kofun populations (*SI Appendix*, Fig. S23). This distinction highlighted the contrasting continental influences that shaped the mainland and the southern islands, revealing a more complex, multilayered formation of the Japanese population than previously recognized.

By utilizing our high-coverage ancient genomes, 67-fold for the Jomon (IY1) and 46-fold for the Yayoi (DO), we identified ancestral components that remained undetected in previous low-coverage or pseudohaploid datasets (Fig. 2*A* and *SI Appendix*, Fig. S7). For the Jomon IY1 individual, the high-coverage genome revealed basal ancestral resources broadly shared across East Asia, Southeast Asia, and Oceania. Furthermore, the diploid sequences facilitated the human leukocyte antigen (*HLA*) genotyping (*SI Appendix*, Figs. S24–S39 and Dataset S10), uncovering specific alleles shared by the present-day Ryukyu and Ainu populations in Japan (*SI Appendix*, Figs. S25 and S26) and allowed the identification of archaic hominin-like segments in the Jomon genome (*SI Appendix*, Fig. S40 and Dataset S11). In addition, our high-coverage Yayoi genome provided the resolution necessary to refine the recently proposed tripartite origins of the Japanese population (7). While previous interpretations relied on low-coverage (0.01 to 0.07-fold) Yayoi samples to suggest that modern Japanese genetic components only appeared during the Kofun period, our data confirmed these elements to already have been established by the Yayoi period. The continuity from the Yayoi period to present-day mainland Japanese, consistent with the earlier observations (28), supported a dual-structure model of the origins of the Japanese population.

Collectively, our results emphasized that the transition to high-coverage ancient genomics could provide a resolution previously unattainable in paleogenomics. The technological shift not only clarified the formation of the Japanese population but also shed light on the origins and evolutionary histories of human populations on a global scale.

## Materials and Methods

### Samples and Sequencing.

This study was approved by the Ethics Committee of Toho University School of Medicine (A23103_A20110_A18099_A18056). We used a Jomon individual IY1 from the Iyai rock shelter site and a Yayoi individual DO from the Doigahama site (*SI Appendix*, see Archeological Information) as samples for whole-genome analyses. Radiocarbon dating showed that IY1 had a calibrated date of 8,300 to 8,200 cal BP (29), which is the later part of the Initial Jomon period. The DO had a radiocarbon date of 2,306 to 2,238 cal BP (18), belonging to the Middle Yayoi period. We extracted DNA from the petrous bones by sampling approximately 130 to 150 mg of bone powder using a sterile electric drill cutter (Dremel). To minimize exogenous DNA contamination, we performed DNA extraction, purification, and NGS library preparation in a dedicated clean room for ancient DNA work, following previously established protocols (16, 17). NGS libraries were prepared for each extract using both single- and double-stranded protocols optimized for Illumina sequencing (Dataset S1). To assess the characteristic ancient DNA damage patterns in the two samples, libraries were constructed without DNA repair treatments. After confirming the presence of postmortem damage, we generated additional libraries using the PreCR Repair Mix (New England BioLabs) to minimize postmortem damage in downstream analyses. We sequenced all libraries on an Illumina HiSeqX platform and used the resulting data for genomic analysis (Dataset S1).

### Read Processing, Mapping, and Ancient DNA Authenticity.

Raw sequencing reads were filtered from each library using FASTP (ver. 0.20.0) (30) and paired adapters, low-quality bases, and reads shorter than 35 bp were removed. The filtered reads from each library were independently mapped to the human reference genome, hg19/GRCh37, using bwa aln (ver. 1.9) (31) with parameters optimized for ancient DNA (-l 1024 -n 0.01 -o 2). PCR and optical duplicates were marked and removed from each library using the MarkDuplicates command of the Picard Toolkit (https://broadinstitute.github.io/picard/). Low-quality mapped reads (<MAPQ30) were removed using SAMtools (ver. 1.9) (32) from the aligned reads.

To evaluate postmortem DNA damages, we confirmed deamination and DNA fragmentation patterns using mapDamage (ver. 2.0.8) (33) with aligned reads from non-repaired NGS libraries. We calculated DNA contamination rates based on definitive haplogroup sites in the mitochondrial genome using MitoSuite (ver.1.0.9) (34, 35), and autosomal DNA contamination of the nuclear X chromosome using ANGSD (ver. 0.939) (36). Finally, we obtained the average depth of coverage across the human genome using SAMtools.

### Biological Sex Estimation, and Maternal and Paternal Lineage Analysis.

To estimate the biological sex of IY1 and DO, we calculated Ry (Y/X + Y) ratios based on the number of reads mapped to the human X and Y chromosomes (37).

To assign the maternal lineages, we estimated the mitochondrial haplogroups of IY1 and DO using MitoSuite (ver. 1.0.9) (34) from the mapped reads. For the male DO sample, we estimated the paternal lineage based on single nucleotide polymorphisms (SNPs) in the non-recombining region (NRY) of the Y chromosome using Y-LineageTracker (ver. 1.3.0) (38). We combined these NRY SNPs with the worldwide Y-lineage dataset (39) and constructed a Y-chromosome phylogenetic tree using Y-LineageTracker (ver. 1.3.0) (38).

### Variant Calling and Population Panel Design.

To minimize postmortem-derived mutations, we trimmed two terminal bases from each read using BamUtil trimBam v1.0.15 (40), and called SNVs on the trimmed BAM files using GATK HaplotypeCaller v3.8.1 (41). To ensure quality, we excluded the sites in masked regions, multiallelic loci, and heterozygotes with alternating allele frequencies <0.2. Following annotation against dbSNP build 138, we integrated variants with reference datasets using PLINK v1.90b4 (42), and calculated coverage using vcftools v0.1.16 (43). We merged our data with the 1240 K SNP panel (44), incorporating worldwide present-day and ancient human samples. To further mitigate mutations derived from DNA damage, we restricted population genomic analyses to transversion sites. We excluded related individuals using READ (45) and KING v2.3.2 (46). The final panel dataset integrated individuals from various regions across Asia (Northeast, East, Central, and South), Oceania, the Middle East, and the Americas (Dataset S2).

### *AMY1* Copy Number Inference From Sequencing Data.

We inferred the copy numbers of the amylase genes in the Yayoi and Jomon individuals from the depth of coverage of the *AMY1* region of the human genome using AMYCN (ver. 2020-03-18) (47) following the workflow used in ancient DNA research (20). We compared the inferred copy numbers with the cumulative distributions of modern hunter-gatherers in East Eurasia, modern Japanese, preagricultural European hunter-gatherers (Labraña 1) (20), and archaic hominins (22, 48).

### Population Genetics, Demographics, and Admixture Analyses.

To place the Jomon (IY1) and Yayoi (DO) individuals within genetic variation across ancient and present-day East Eurasians, we first analyzed the genome-wide SNP data using PCA implemented in smartpca (EIGENSOFT ver. 7.2.1) (49). Prior to PCA, we performed linkage disequilibrium (LD) pruning in PLINK (ver. 1.90) (42) using “--indep-pairwise 50 10 0.1.” We disabled outlier removal (“numoutlieriter” = 0), applied normalization options, and used lsqproject so that no sample was automatically excluded.

To summarize the ancestry components and assess how Japanese archipelago samples relate to other ancient and present-day populations, we performed unsupervised clustering using Admixture (ver. 1.3.0) (50). We explored *K* = 2 to 17 with ten independent runs and fivefold cross-validation, repeated each *K* ten times to confirm convergence, and summarized the replicate solutions with CLUMPAK (ver. 1.1.2) (51).

We evaluated the genetic affinities and population relationships using allele-sharing statistics and tree-based modeling. We computed the outgroup-*f3* statistics using qp3pop (AdmixTools; ver. 980) (52), quantifying the shared genetic drift between populations X and Y relative to an outgroup (Mbuti). In addition, maximum-likelihood population trees were inferred with TreeMix (ver. 1.13) (53) using a subset of ancient and present-day individuals across East Eurasia, Siberia, Oceania, and ancient North America (Ust_Ishim, Sunghir_UP, Yana_UP, MA1, Tianyuan, Papuan, Onge, USR, Jomon, Shamanka_EN, Chokhopani, Ami, CHB, Yayoi [DO], JPT, Devil’s Cave_N, and Kolyma). Mbuti was used as the outgroup and models that allowed 0 to 5 migration edges (m = 0 to 5) were tested.

To reconstruct the demographic histories of the two high-coverage ancient genomes, we generated whole-genome diploid consensus sequences from the mapped reads using bcftools (ver. 1.9) (54) and inferred long-term changes in the effective population size using PSMC (ver. 0.6.5-r67) (55). To assess robustness and reduce spurious peak detection, we ran 100 bootstrap replicates and tested alternative first time-slice schemes (4, 2 + 2, and 1 + 1 + 1 + 1), as recommended previously (56). To capture more recent dynamics since the LGM, we inferred the population size history using SMC + + (ver. 1.15.2) (57) for unphased genomes. In addition, we estimated runs of homozygosity (ROH) using the hapROH program (58). We applied hapROH (version 1.0) to each individual using the genotype datasets. Individuals with insufficient genome coverage for reliable ROH inference were excluded from the analysis.

Finally, to characterize the admixture processes relevant to the formation of present-day Japanese populations, we estimated admixture timing using ALDER (ver. 1.0.3) (59). We used modern Japanese as the target and modeled ancestry sources using IY1 (Jomon), DO (Yayoi), and Han (mainland China). Further, we evaluated plausible admixture histories using graph- and mixture-based frameworks in AdmixTools: qpGraph (ver. 7.0.2) (60, 61) to test the explicit Admixture graphs (using Dinka, Malta, Onge, MA1, Shamanka, Alaska_LP, USA_Ancient_Beringian, Chokhopani, Jomon Iyai, and Yayoi Doigahama), and qpAdm/qpWave (ver. 7.0.2) (60, 61) to estimate mixture proportions and test the minimum number of ancestry streams. For qpAdm/qpWave, we fixed Jomon as a source for modern Japanese/Yayoi/Kofun and tested alternative continental sources using the following outgroups: Mbuti, Tianyuan, Papuan, Onge, Nganasan, Yamnaya_Samara, DevilsCave_N, and Ust_Ishim.

### *HLA* Genotyping.

We determined the human leukocyte antigen (*HLA*) type of IY1 and DO using shotgun sequencing reads. We performed *HLA* typing based on 4-digit alleles using xHLA (ver.4.0.3) (62). Finally, we retrieved the frequencies of inferred *HLA* typing in the local population from the allele frequency net database (http://www.allelefrequencies.net/) (63).

### Inference of Introgressed Fragments From Archaic Hominins.

To infer the ancestry fragments from archaic hominins (Neanderthal and Denisovan) across each genomic position in the Jomon and Yayoi individuals, we attempted to fit the allele frequency by comparing it with reference sources based on a Hidden Markov Model (64) (https://github.com/BenjaminPeter/admixfrog). The reference resources used were the Neanderthals and Denisovans from Vindija, and Altai and Yoruba from the African population. We set the “--c0” option, assuming a low level of contamination in our dataset.

## Supplementary Material

Appendix 01 (PDF)

Dataset S01 (XLSX)

Dataset S02 (XLSX)

Dataset S03 (XLSX)

Dataset S04 (XLSX)

Dataset S05 (XLSX)

Dataset S06 (XLSX)

Dataset S07 (XLSX)

Dataset S08 (XLSX)

Dataset S09 (XLSX)

Dataset S10 (XLSX)

Dataset S11 (XLSX)

## Data Availability

The genomic variant data generated in this study were deposited in EMBL-EBI under the accession number PRJEB84363 (65). Sequencing data from the 1000 Genomes Project and the Human Genome Diversity Project (HGDP) were downloaded from The International Genome Sample Resource (https://www.internationalgenome.org) (66). Published ancient genomic data were obtained from The Allen Ancient DNA Resource dataset (AADR) version 54.1. p1 (https://dataverse.harvard.edu/dataset.xhtml?persistentId=doi:10.7910/DVN/FFIDCW) (67).
