# Supplementary material for: High-coverage ancient genomes reveal divergent population histories and prehistoric starch-related genetic variation in Japan
Source: Proc Natl Acad Sci U S A. 2026 Jul 23;123(30):e2606162123. doi: 10.1073/pnas.2606162123 (PMC13416418; doi:10.1073/pnas.2606162123)
Supplement: Supplementary file 1 — Appendix 01 (PDF) [file pnas.2606162123.sapp.pdf]

PNAS

Supporting Information for
High-coverage ancient genomes reveal divergent population histories and prehistoric starch-related genetic variation in Japan

Koji Ishiya[a, b, c, 1, 2], Fuzuki Mizuno[d, 1], Jun Gojobori[e, 2], Masahiko Kumagai[f, 2], Yasuhiro Taniguchi[g], Osamu Kondo[c], Masami Matsushita[h], Takayuki Matsushita[h], Li Wang[i, 2], Kunihiko Kurosaki[d], Shintaroh Ueda[c, d]

[a]Sapiens Life Sciences, Evolution and Medicine Research Center, Kanazawa University; Kanazawa, 920-8640, Japan.
[b]Institute for the Study of Ancient Civilizations and Cultural Resources, Kanazawa University; Kanazawa, 920-1192, Japan.
[c]Department of Biological Sciences, Graduate School of Science, The University of Tokyo; Bunkyo-ku, 113-8654, Japan.
[d]Department of Legal Medicine, Toho University School of Medicine; Ōta-ku, 143-0015, Japan.
[e]Research Center for Integrative Evolutionary Science, The Graduate University for Advanced Studies (SOKENDAI); Hayama, 240-0193, Japan.
[f]Research Center for Advanced Analysis, National Agriculture and Food Research Organization; Tsukuba, 305-8517, Japan.
[g]Department of Archaeology, Faculty of Letters, Kokugakuin University; Shibuya-ku, 150-8440, Japan.
[h]The Doigahama Site Anthropological Museum; Shimonoseki, 759-6121, Japan.
[i]Department of Evolutionary Studies of Biosystems, The Graduate University for Advanced Studies (SOKENDAI); Hayama, 240-0193, Japan.

[1]These authors contributed equally to this work.
[2]Corresponding authors: Koji Ishiya, Jun Gojobori, Masahiko Kumagai, Li Wang
Email: ishiya@staff.kanazawa-u.ac.jp (to. K.I.), gojobori_jun@soken.ac.jp (to. J.G.), kumagai.masahiko243@naro.go.jp (to. M.K.), 2234593631@qq.com (to. L.W.)

This PDF file includes:

Supporting Information Text
Figures S1 to S40
Datasets S1 to S11 (separate file)
SI References

Supporting Information Text

Archeological information

We investigated the Iyai rock shelter site, located on the edge of the Jo-shin-etsu mountains between the Shinano and Tone Rivers, in the center of Honshu, Japan (Naganohara-cho, Gunma Prefecture; 36°33'28" N, 138°38'50" E) (Fig. 1). The shelter consists of a rock wall composed of welded tuff. A previous report has identified this site as spanning the Initial Jomon to the Late Yayoi period, with an open-sloping terrace extending from inside the shelter to the front area (1). Ash-rich soil layers had accumulated within the shelter and were protected from rainwater. We found human remains in soil layers with high concentrations of calcite derived from wood ash. We discovered the Iyai1 (IY1) individual in the summer of 2015 and excavated its nearly complete skeleton in 2016 (2). Researchers at the University of Tokyo authenticated the remains and estimated their age using radiocarbon dating calibrated with the IntCal13 curve, determining an age of 8300–8200 calBP (2).

We also examined the Doigahama site, an early to middle Yayoi cemetery located on Doigahama beach in southwestern Honshu, Japan (Shimonoseki City, Yamaguchi Prefecture; 34°17'26" N, 130°53'19" E) (Fig. 1). This site lies on a dune composed of windblown sand, including abundant shell fragments and mineral particles. The lime content of the shells mixed with sand helped preserve bones. Archaeological excavations have uncovered more than 300 human skeletal remains at this site. In 1994, a staff member of the Hohoku-cho Board of Education in Yamaguchi Prefecture discovered the skeletal remains of the individual referred to as DO (DH-S01) at the Doigahama site in Shimonoseki City, Yamaguchi Prefecture, Japan. The Doigahama Anthropological Museum authenticated the ancient human remains. Paleo Labo Co., Ltd. conducted radiocarbon dating using the IntCal13 calibration curve, estimating the age at 2306–2238 calBP (3). The remains are currently hosted at the Doigahama Anthropological Museum.

Figures

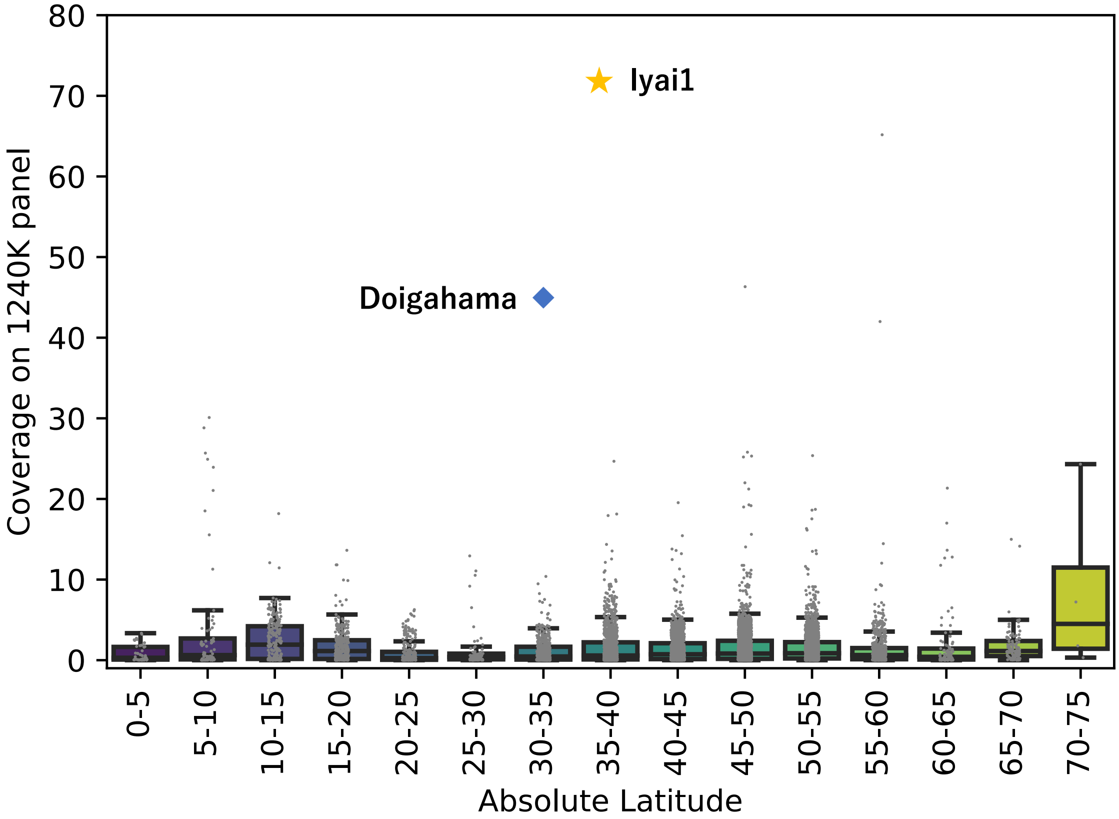

Fig. S1. Comparison of SNP coverages across published ancient samples.

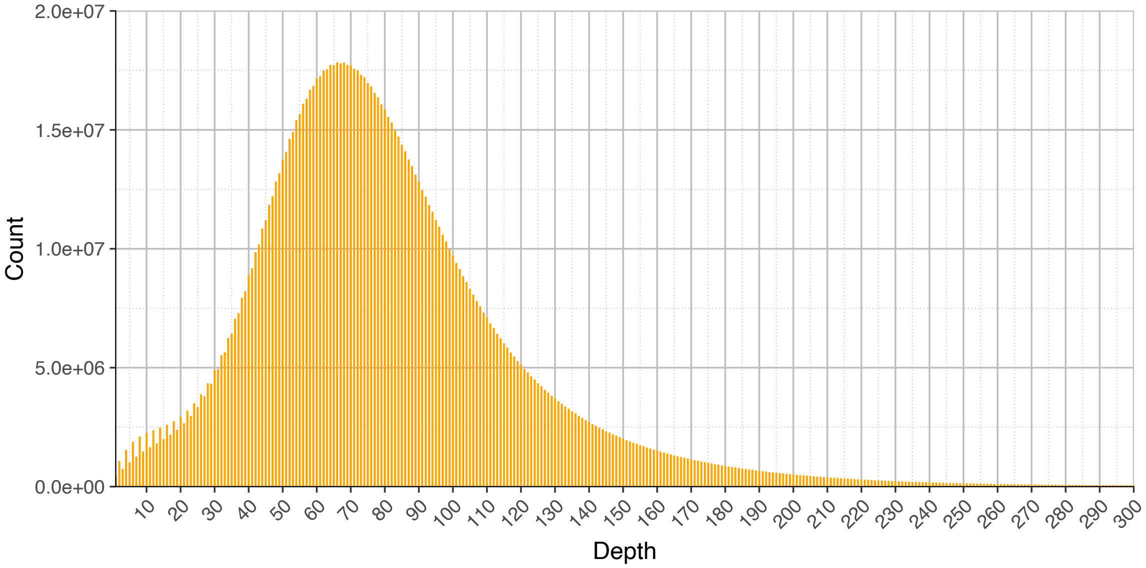

Fig. S2. Distribution of sequencing read coverage of the Initial-Jomon sample IY1.

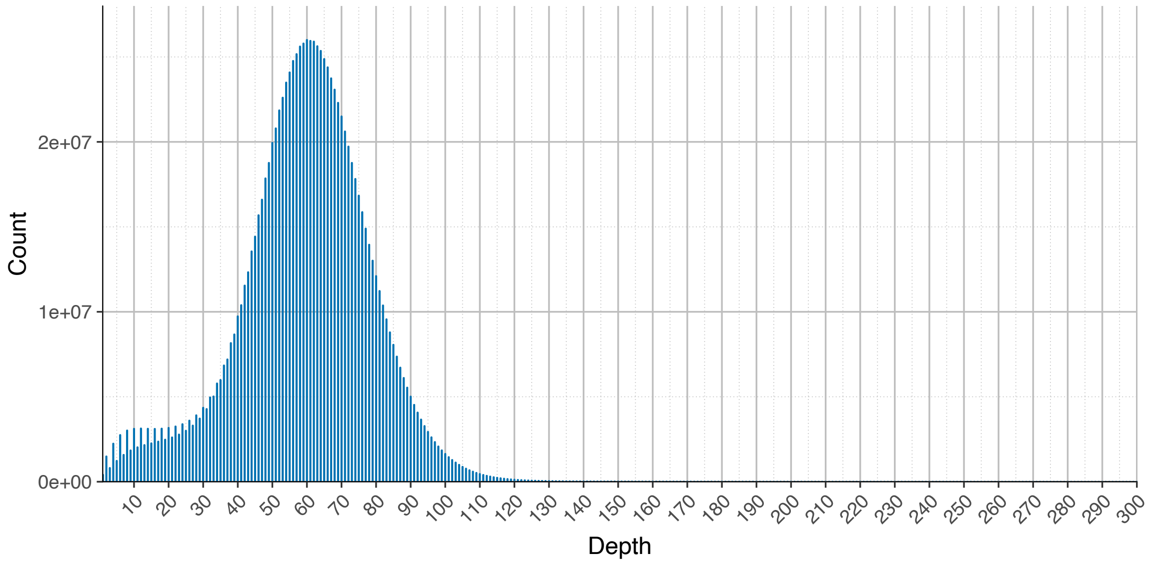

Fig. S3. Distribution of sequencing read coverage of the Middle-Yayoi sample DO.

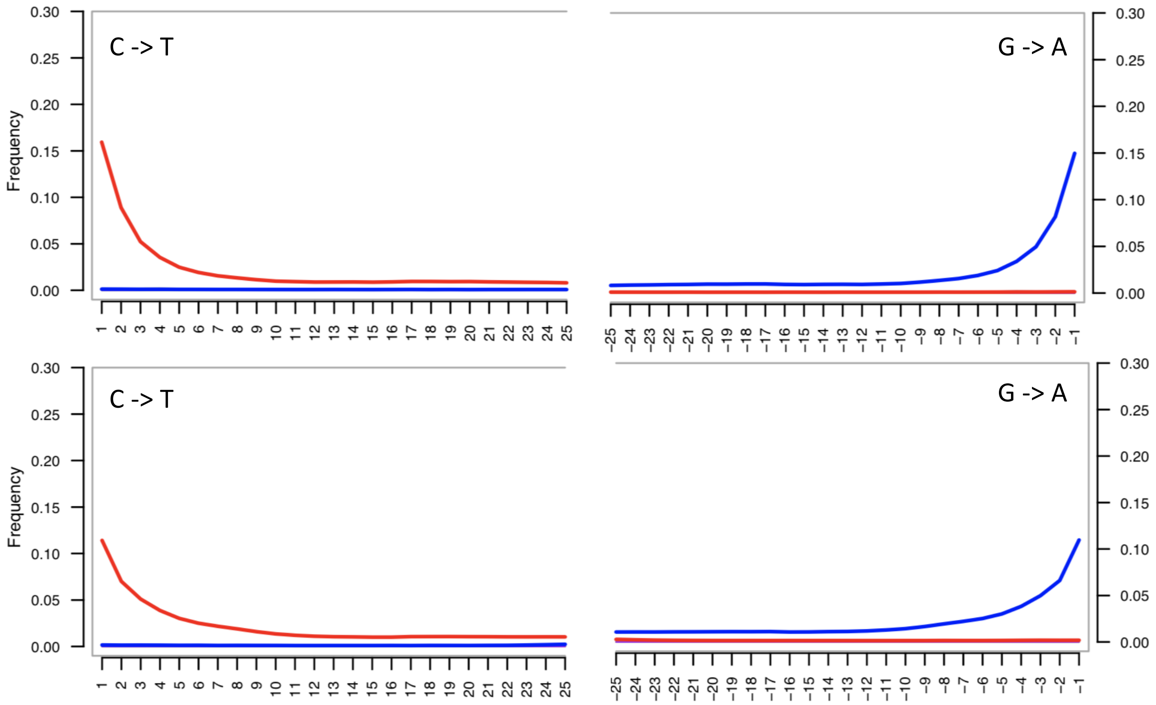

Fig. S4. Deamination patterns on mapped reads from IY1 (upper) and DO (lower).

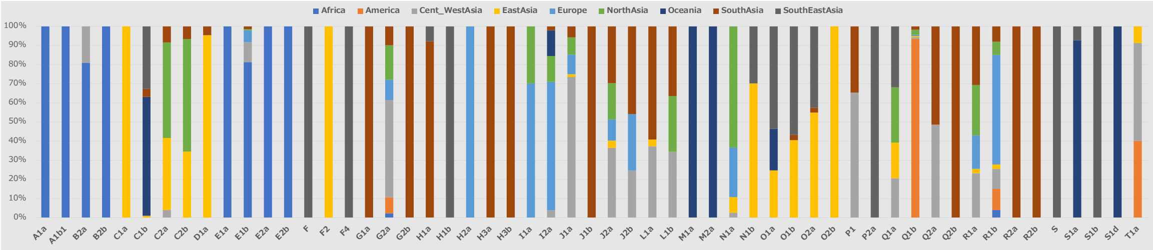

Fig. S5. Proportion of Y-chromosome haplogroups in the worldwide populations (n=1,195).

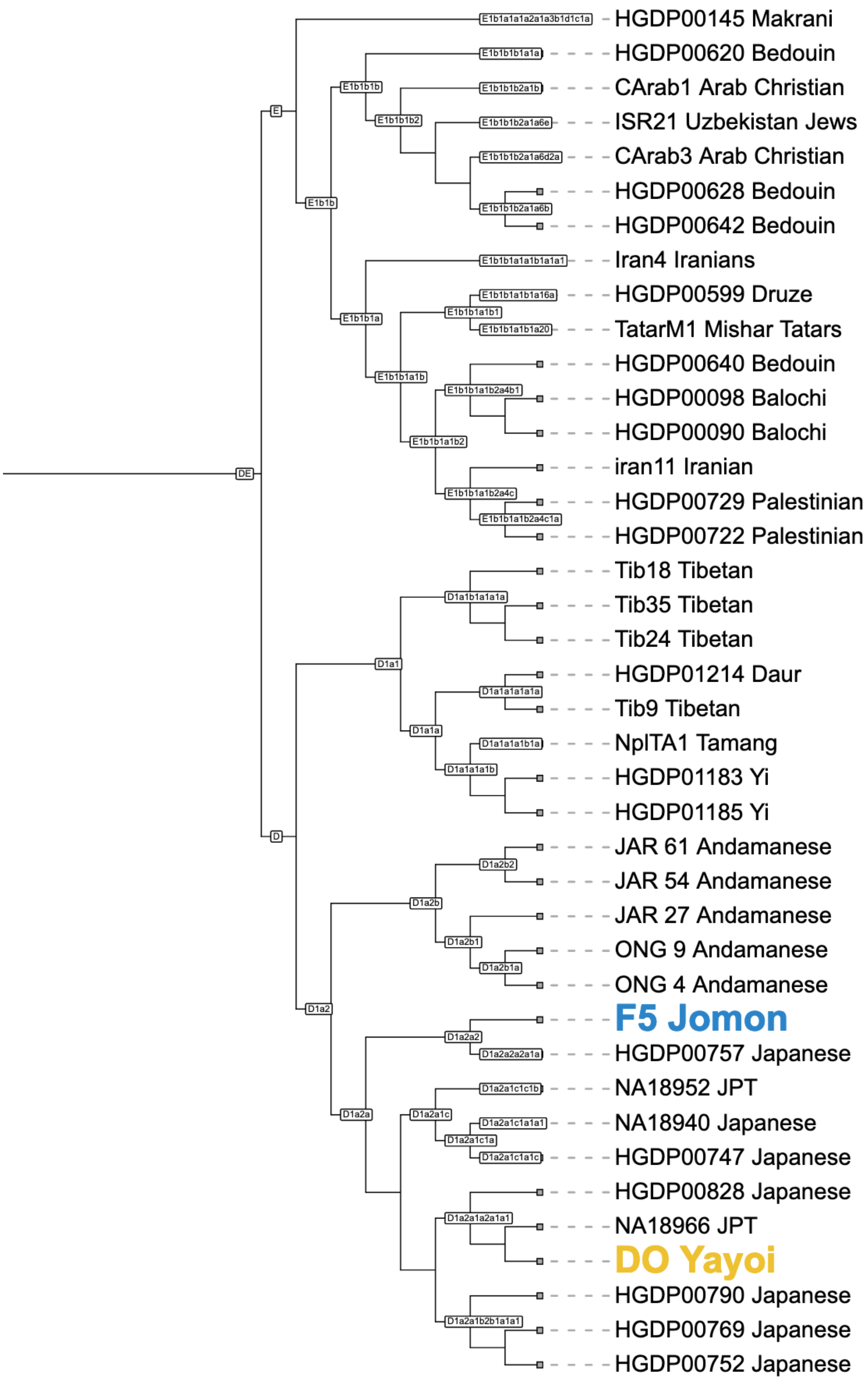

Fig. S6. Phylogenetic relationships based on Y-chromosome genome wide SNPs.

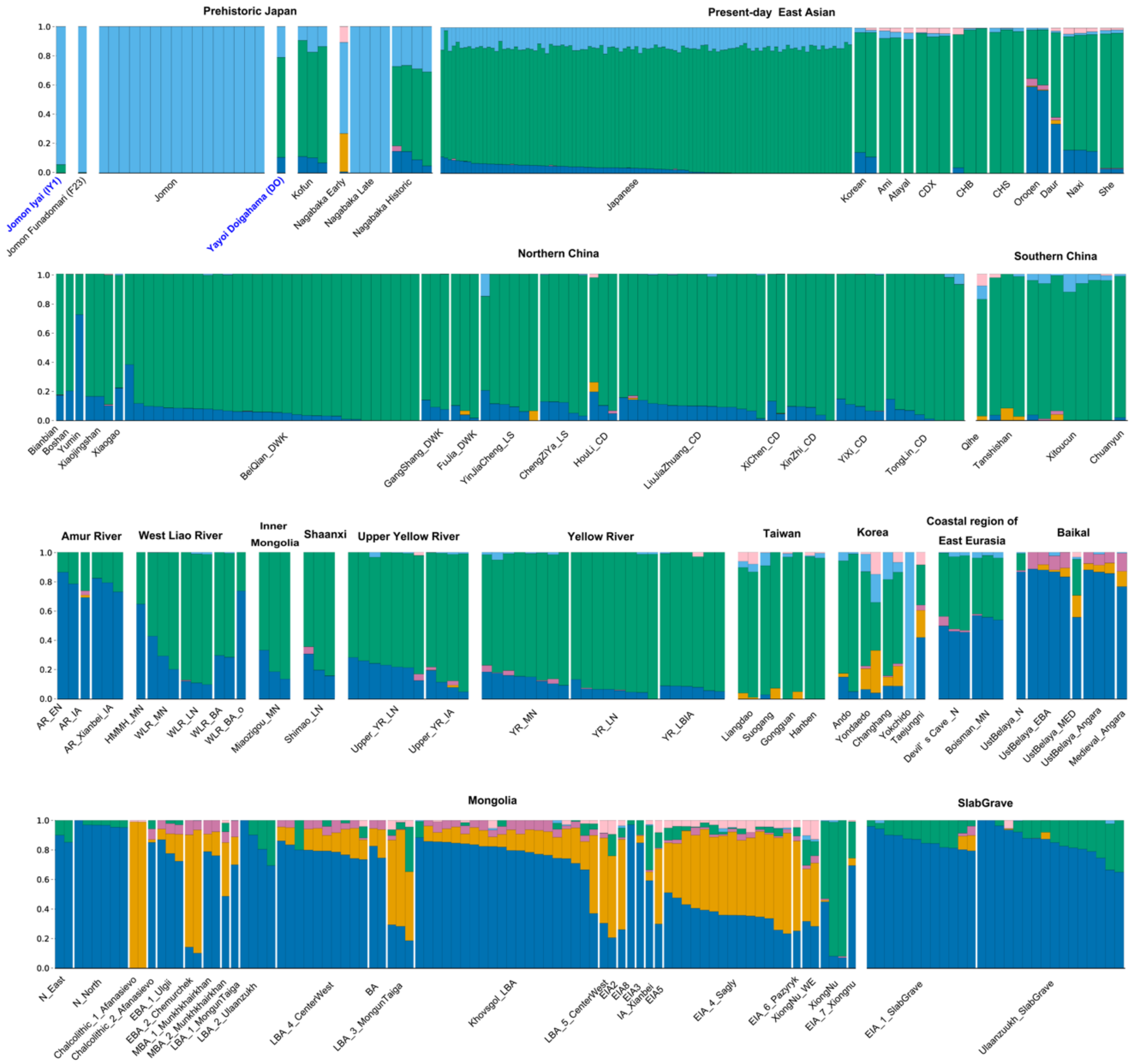

Fig. S7. ADMIXTURE analysis of East Eurasian continental samples. This panel presents East Eurasian populations based on ADMIXTURE analysis at K = 6. Newly sequenced individuals appear in blue. The analysis tested ancestral components from K = 2 to K = 17, with K = 6 producing the lowest cross-validation error (Fig. S8). The color scheme for inferred ancestry components is as follows: P1 (blue), P2 (orange), P3 (yellow), P4 (green), P5 (light blue), and P6 (red).

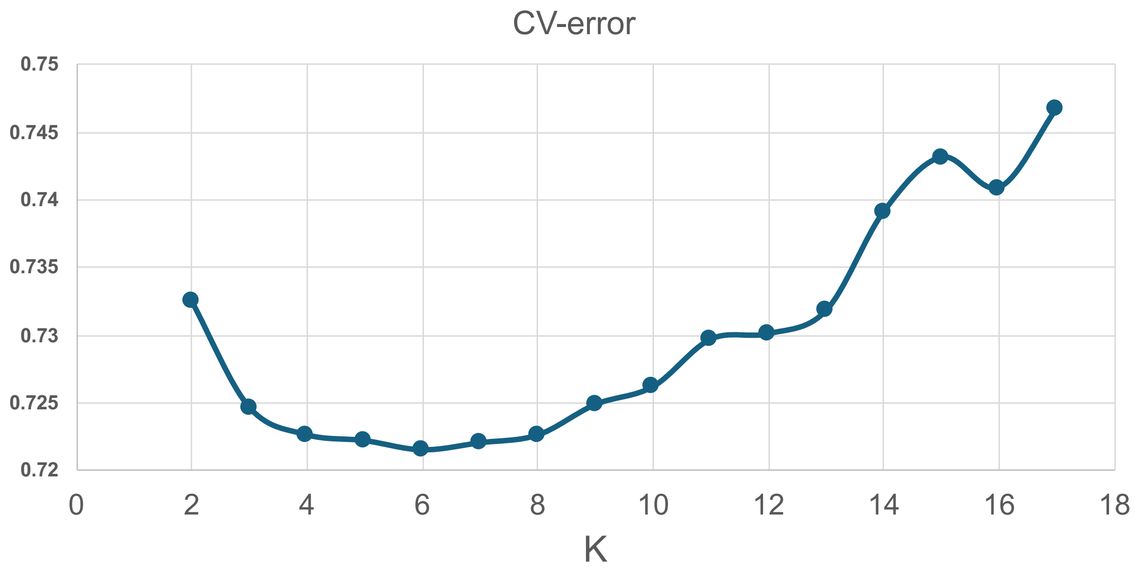

Fig. S8. Phylogenetic relationships based on Y-chromosome genome wide SNPs.

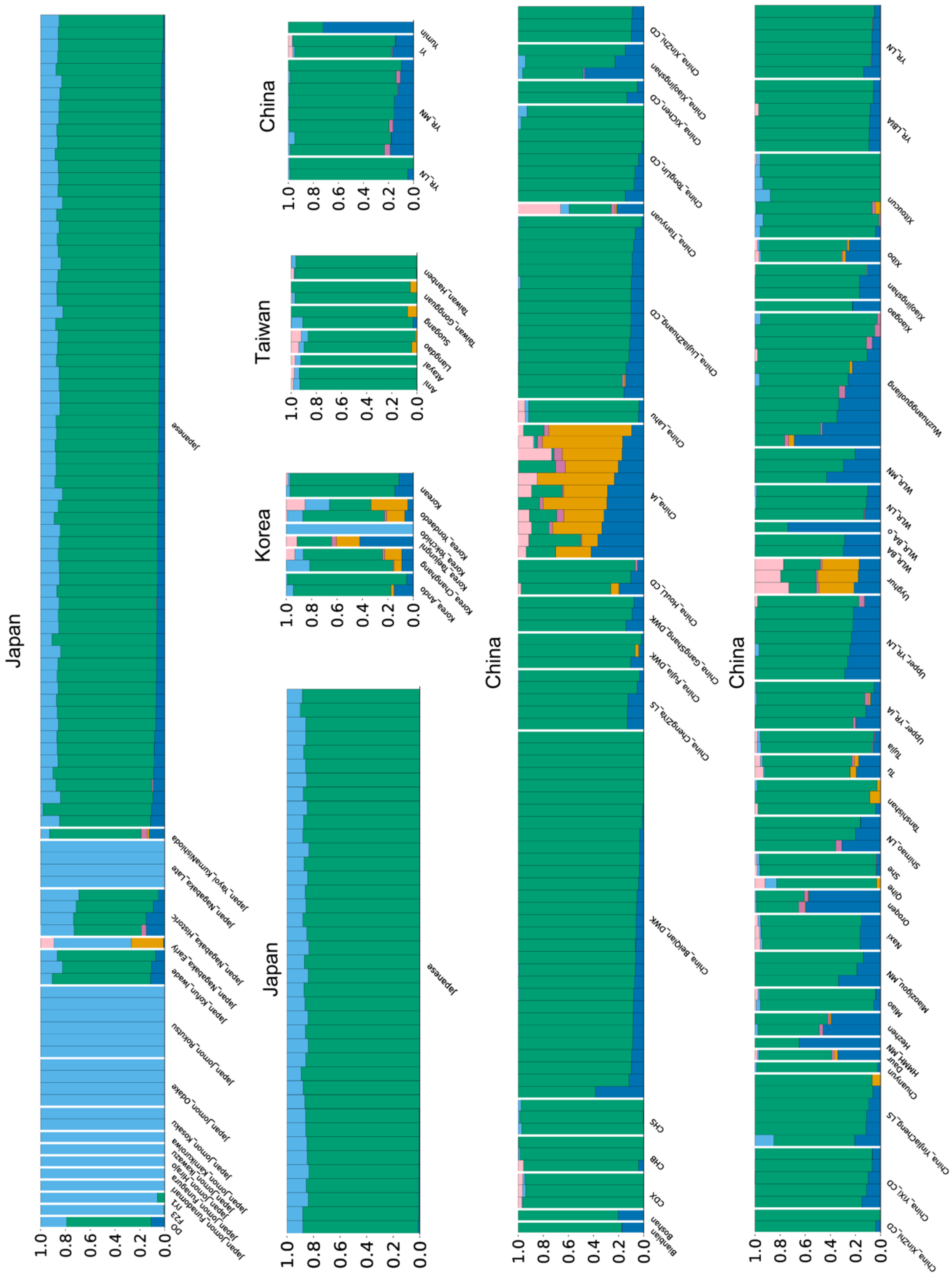

Fig. S9 (1/8). Result of ADMIXTURE analysis. This panel shows the results of East Asia populations at K = 6, which yielded the lowest cross-validation error. Each bar represents the proportion of inferred ancestral components for an individual.

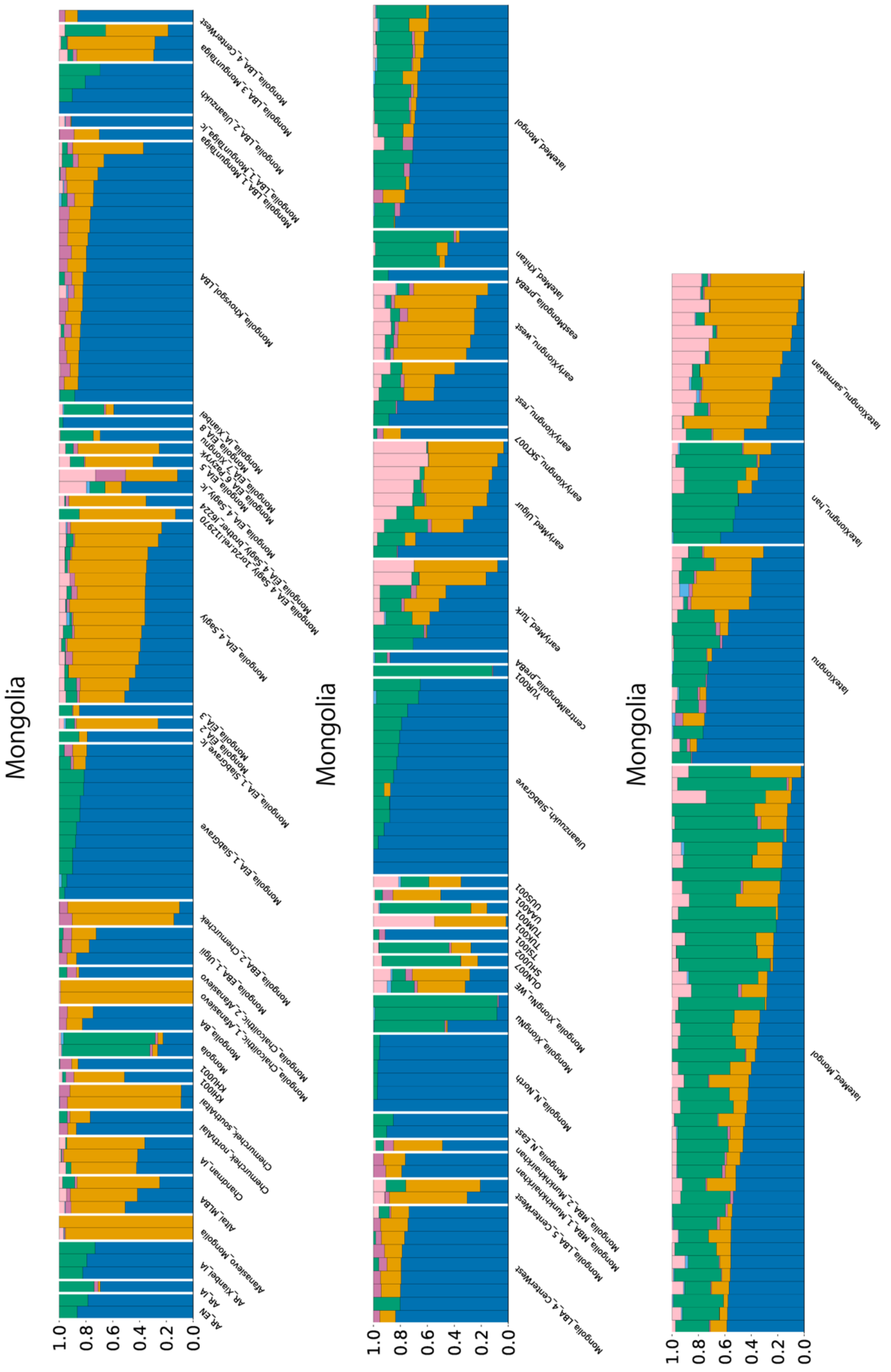

Fig. S9 (2/8). Result of ADMIXTURE analysis. This panel shows the results of Mongolia populations at K = 6, which yielded the lowest cross-validation error. Each bar represents the proportion of inferred ancestral components for an individual.

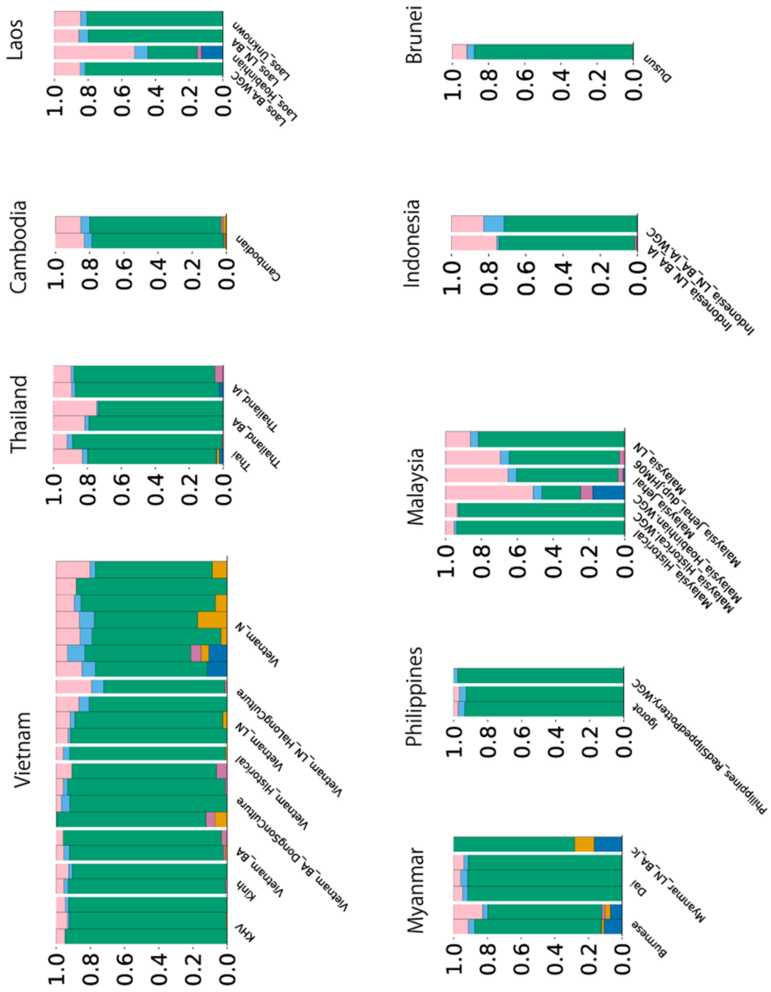

Fig. S9 (3/8). Result of ADMIXTURE analysis. This panel shows the results of Southeast Asia populations at K = 6, which yielded the lowest cross-validation error. Each bar represents the proportion of inferred ancestral components for an individual.

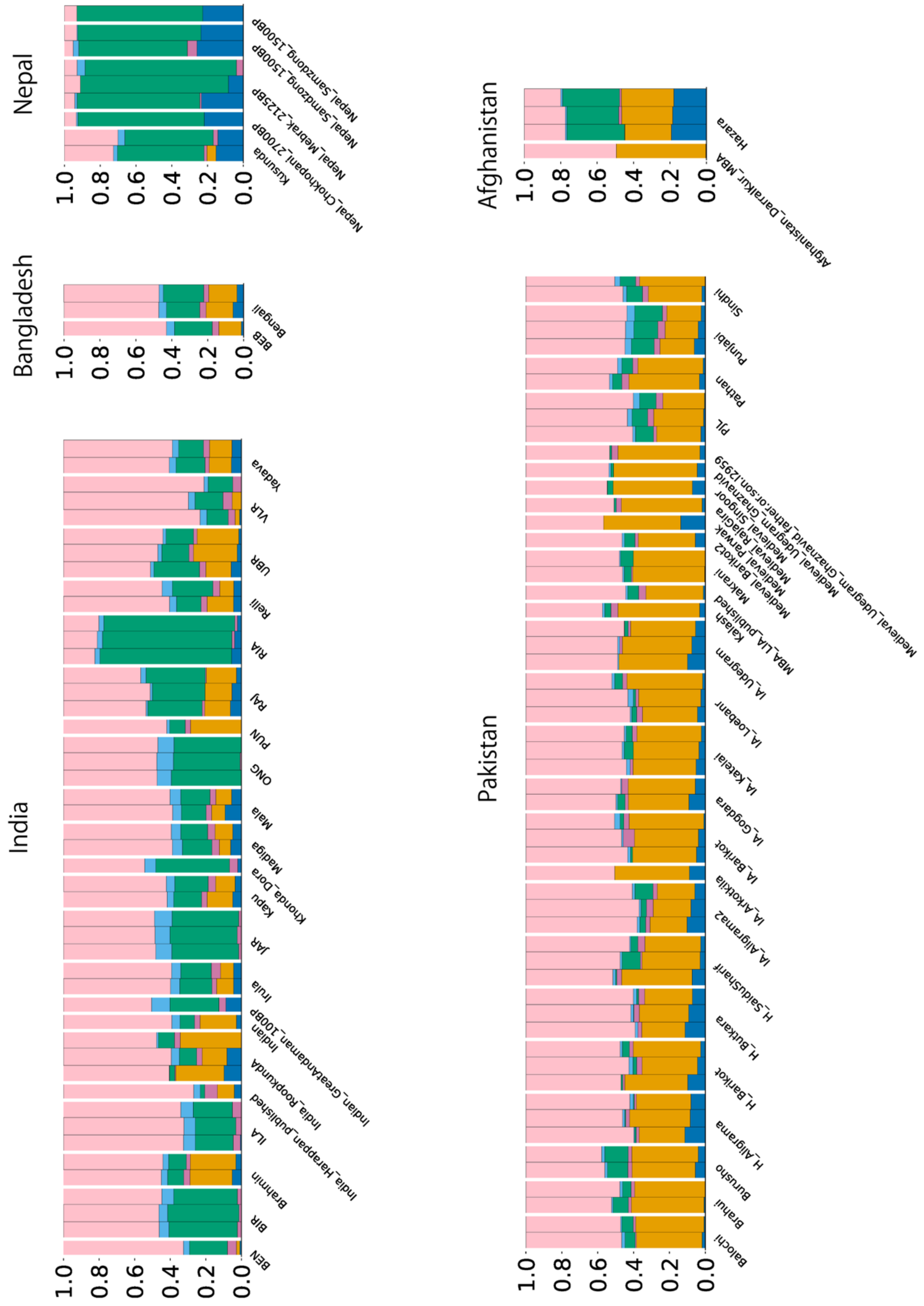

Fig. S9 (4/8). Result of ADMIXTURE analysis. This panel shows the results of South Asia populations at K = *6*, which yielded the lowest cross-validation error. Each bar represents the proportion of inferred ancestral components for an individual.

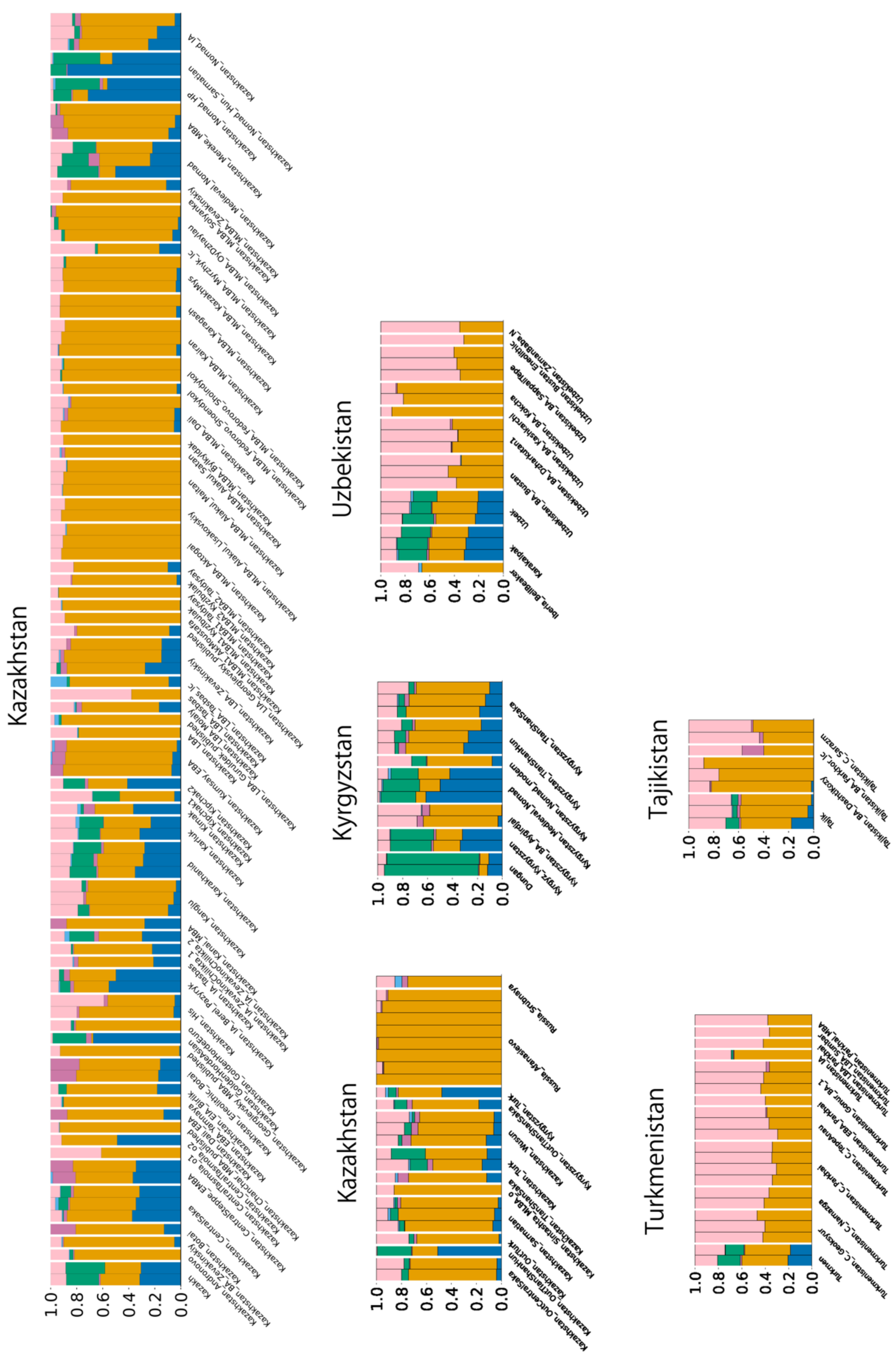

Fig. S9 (5/8). Result of ADMIXTURE analysis. This panel shows the results of Central Asia populations at K = 6, which yielded the lowest cross-validation error. Each bar represents the proportion of inferred ancestral components for an individual.

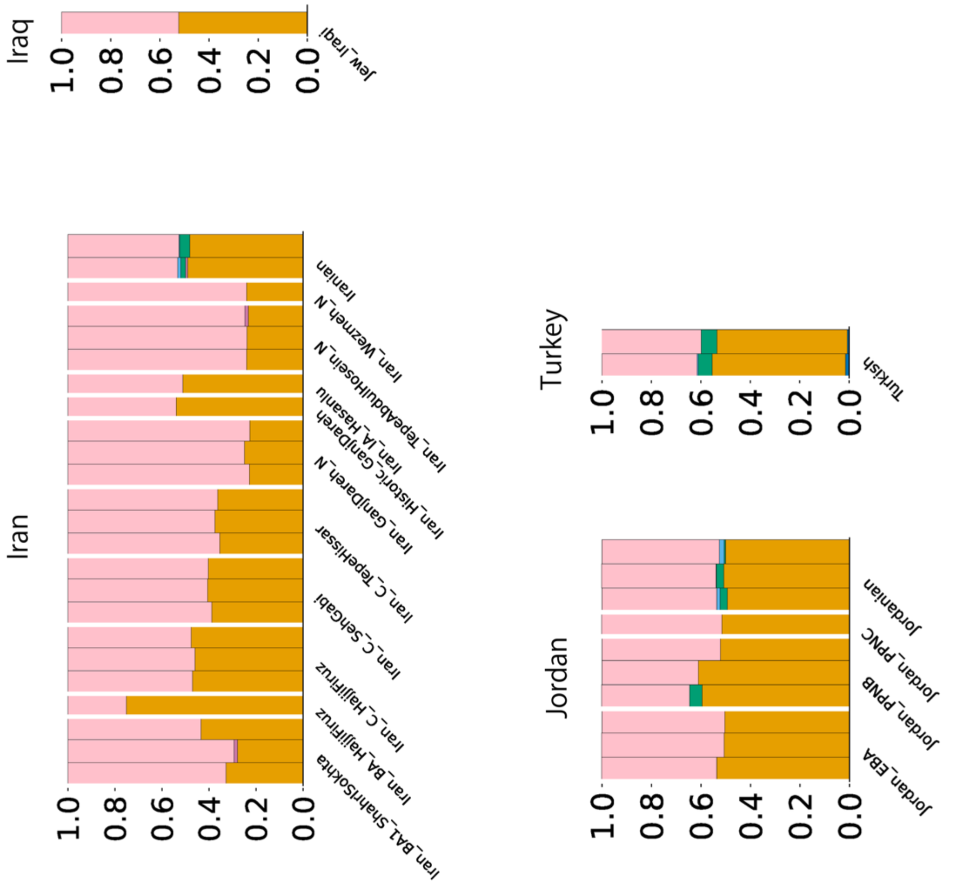

Fig. S9 (6/8). Result of ADMIXTURE analysis. This panel shows the results of Middle East and West Asia populations at K = 6, which yielded the lowest cross-validation error. Each bar represents the proportion of inferred ancestral components for an individual.

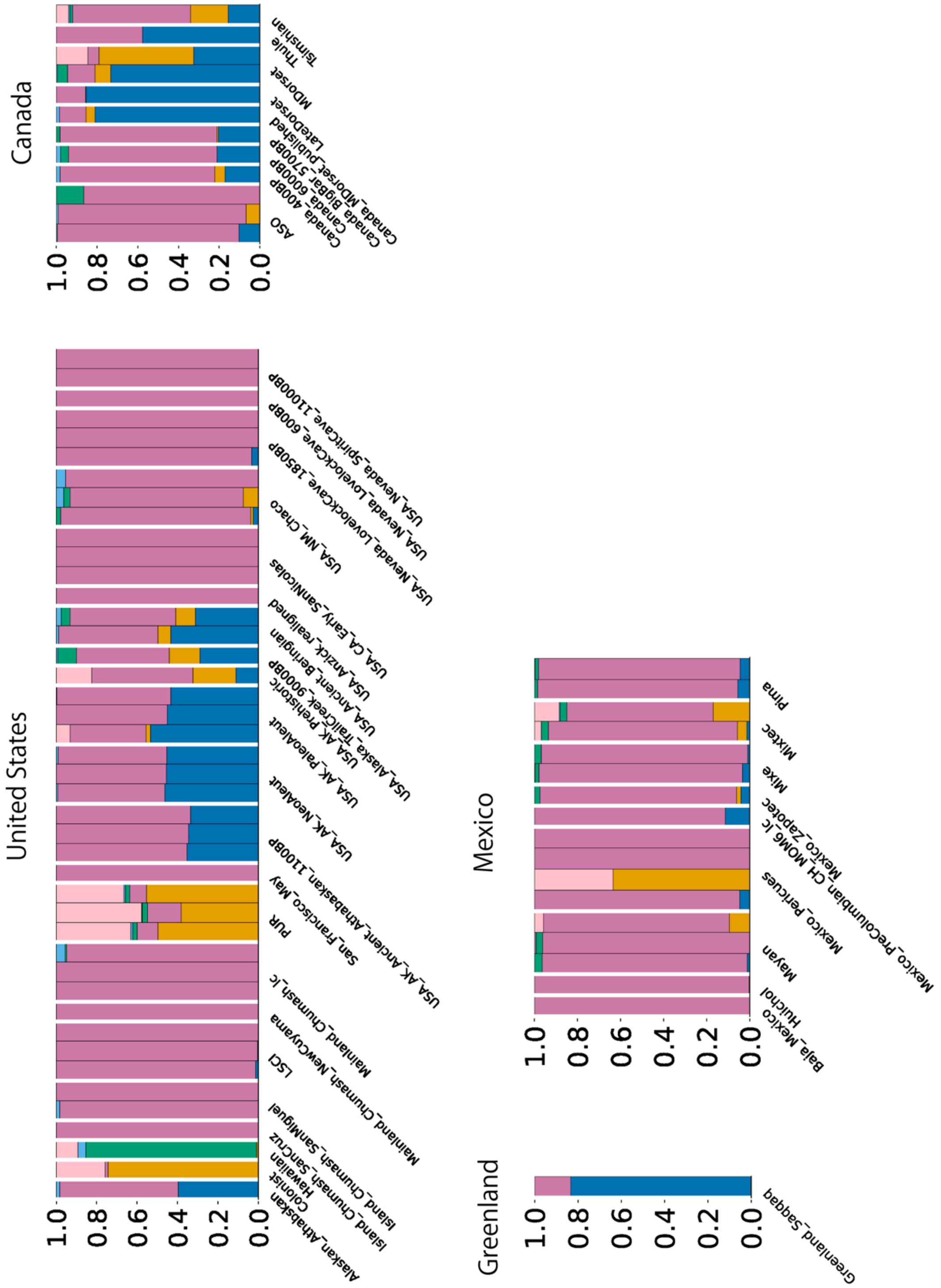

Fig. S9 (7/8). Result of ADMIXTURE analysis. This panel shows the results of North America populations at K = 6, which yielded the lowest cross-validation error. Each bar represents the proportion of inferred ancestral components for an individual.

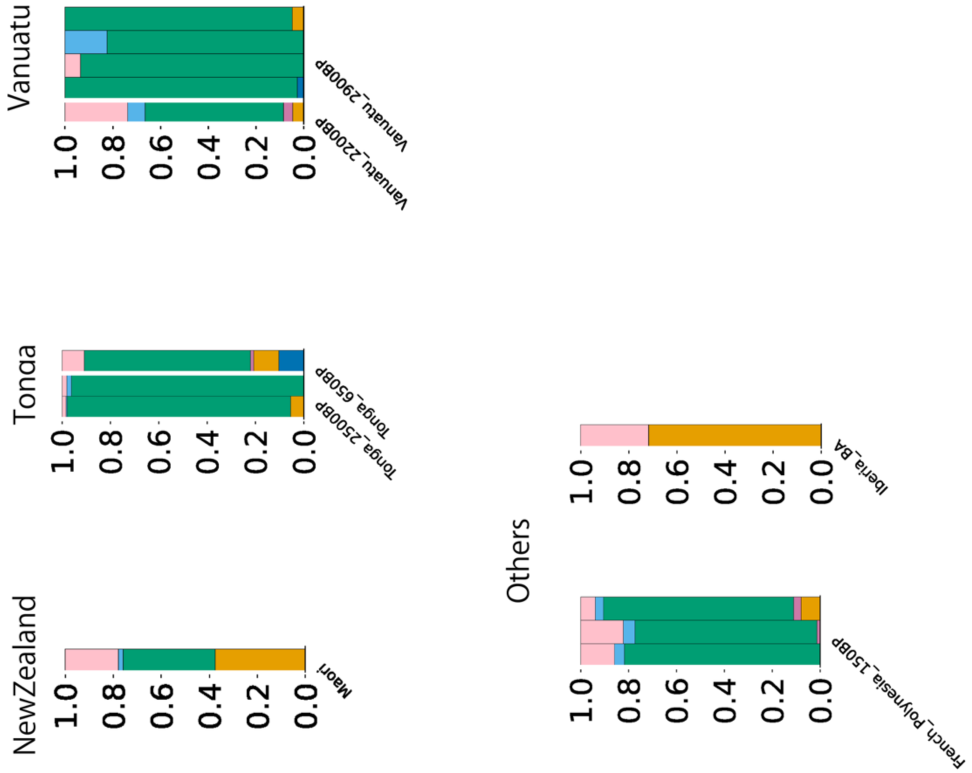

Fig. S9 (8/8). Result of ADMIXTURE analysis. This panel shows the results of Oceania and other region populations at *K* = 6, which yielded the lowest cross-validation error. Each bar represents the proportion of inferred ancestral components for an individual.

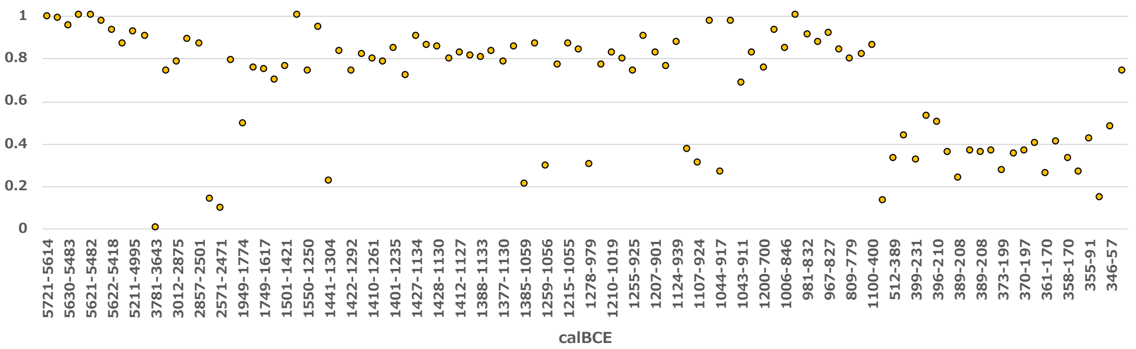

Fig. S10. The frequency of the ancestral component (P1) observed among ancient Mongolia samples in ADMIXTURE analysis. The horizontal axis shows the calibrated radiocarbon date (calBCE) of the sample and the frequency of the ancestral component (P1) of each individual on the horizontal axis.

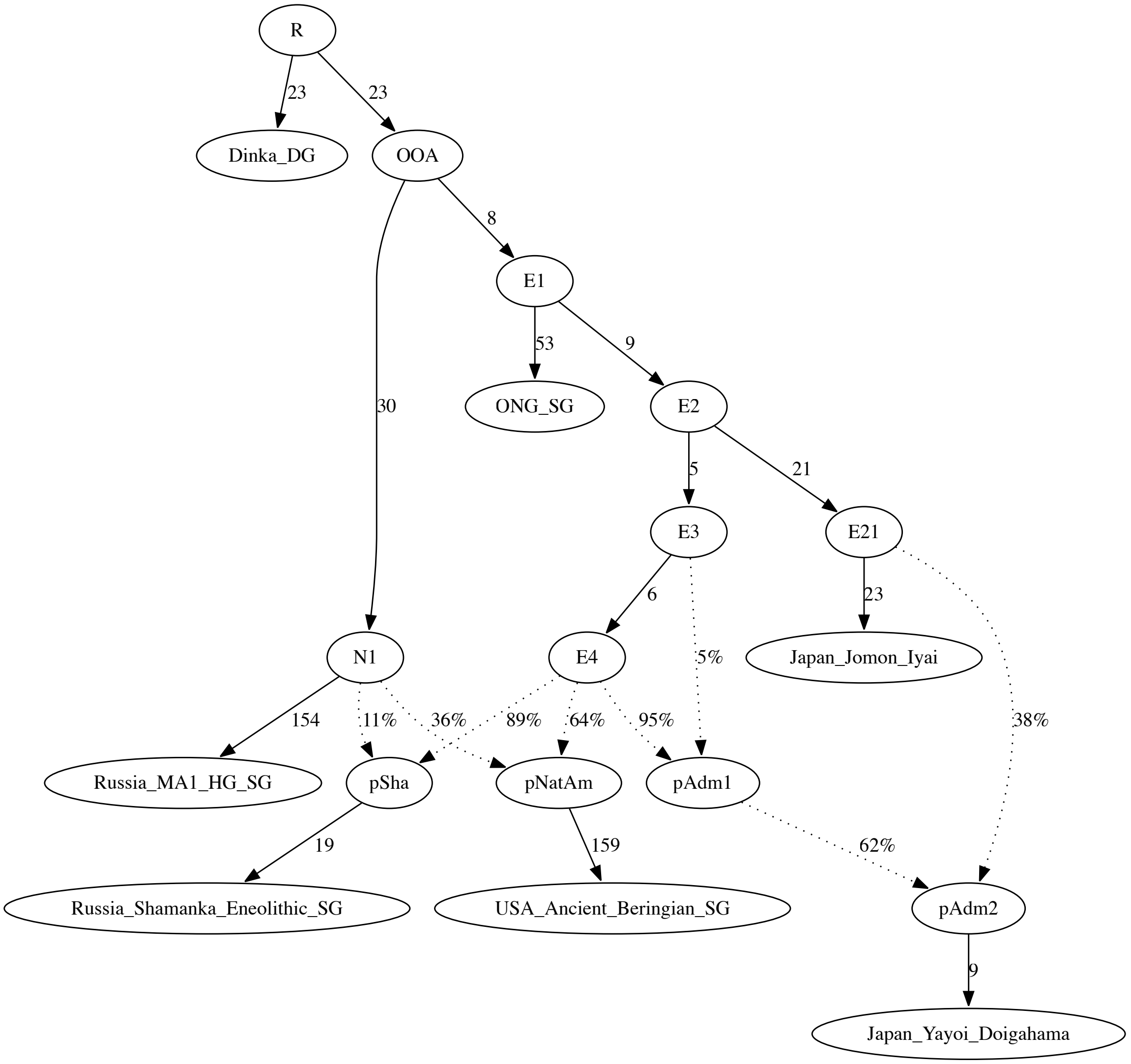

Fig. S11. An admixture modeling under possible ancestral sources (*Z*=-2.340).

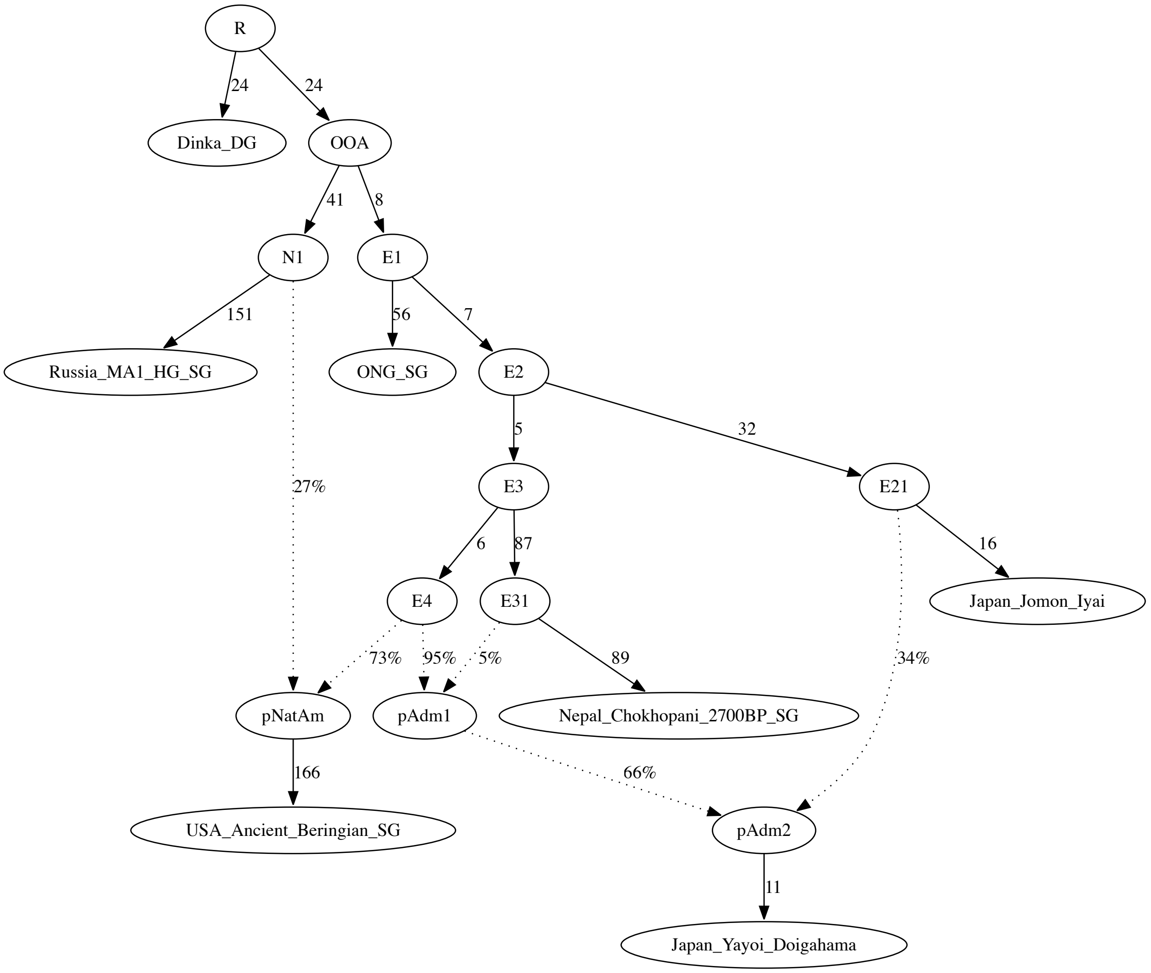

Fig. S12. An admixture modeling under possible ancestral sources (*Z*=-3.120).

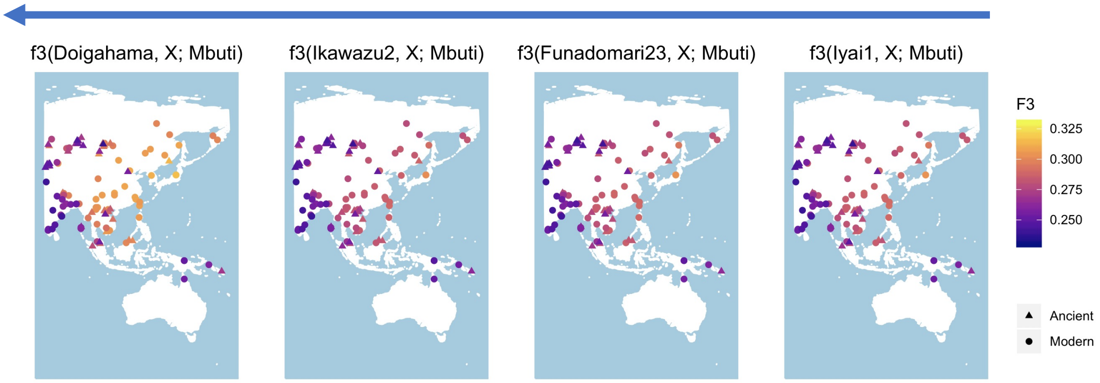

Fig. S13. Transformation of genetic affinities among surrounding East Eurasian populations. This figure presents a summary of the *f3* values for the representative ancient human genomes (Iyai1 (DO), Funadomari23 (F23), Ikawazu2 (IK002), and Doigahama (DO)) from the initial Jomon to Yayoi periods in the Japanese archipelago in relation to the surrounding regional populations. The colors correspond to the *f3* values, with brighter, yellow-colored points indicating higher genetic affinity.

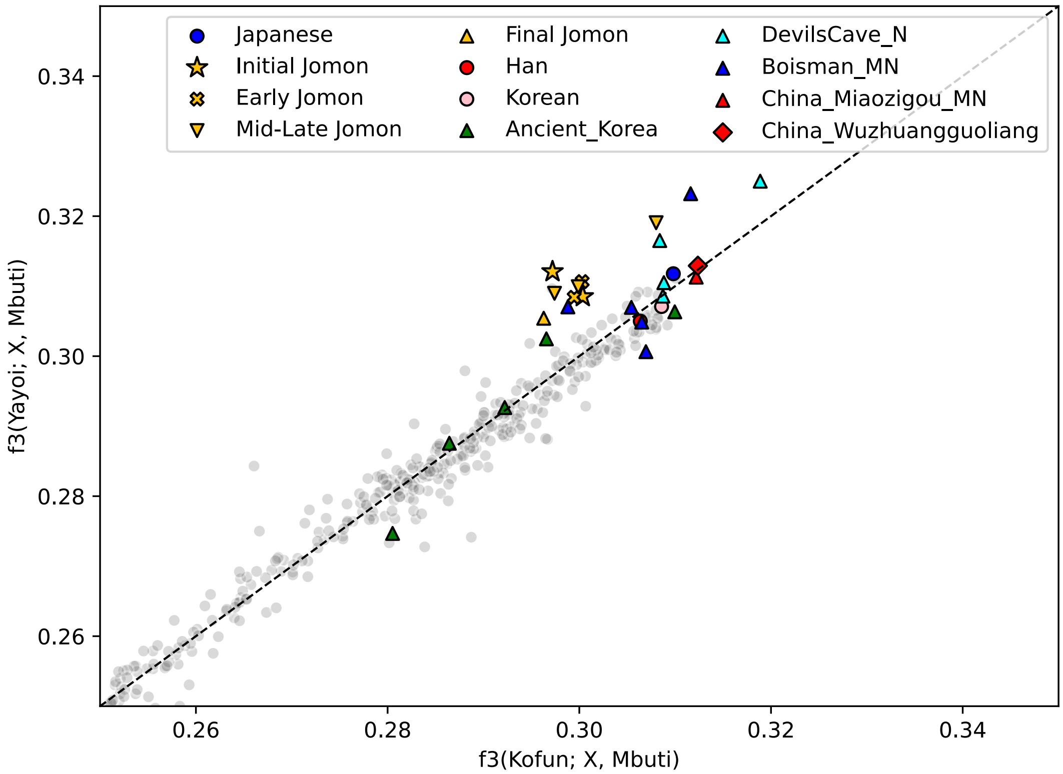

Fig. S14. A comparison of outgroup-*f3* statistics with the Yayoi and Kofun individuals against East Asians.

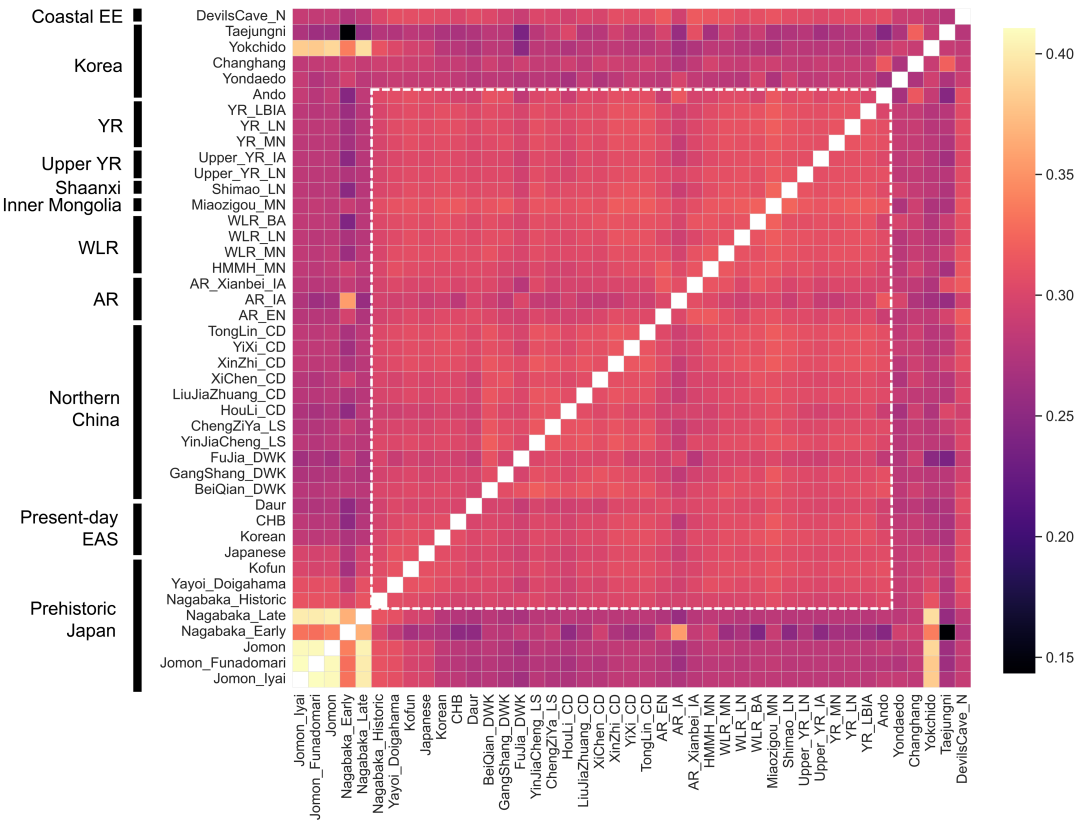

Fig. S15. Heatmap of pairwise outgroup-*f3* statistics among ancient and present-day East Asian populations. This figure illustrates the genetic affinity between populations, based on pairwise outgroup-*f3* statistics of the form *f3*(Mbuti; X, Y), using Mbuti as the outgroup. The heatmap color intensity represents the degree of their genetic affinities. Population exhibiting similar levels of affinity are outlined with white wavy lines. Key individuals and representative populations for geographic and cultural regions, such as Prehistoric Japan, Present-day EAS (East Asia), Northern China, AR (Amur River), WLR (West Liao River), Shaanxi, Inner Mongolia, Upper YR (Upper Yellow River), YR (Yellow River), Korea, and Coastal EE (Coastal East Eurasia), are labeled in bold black text.

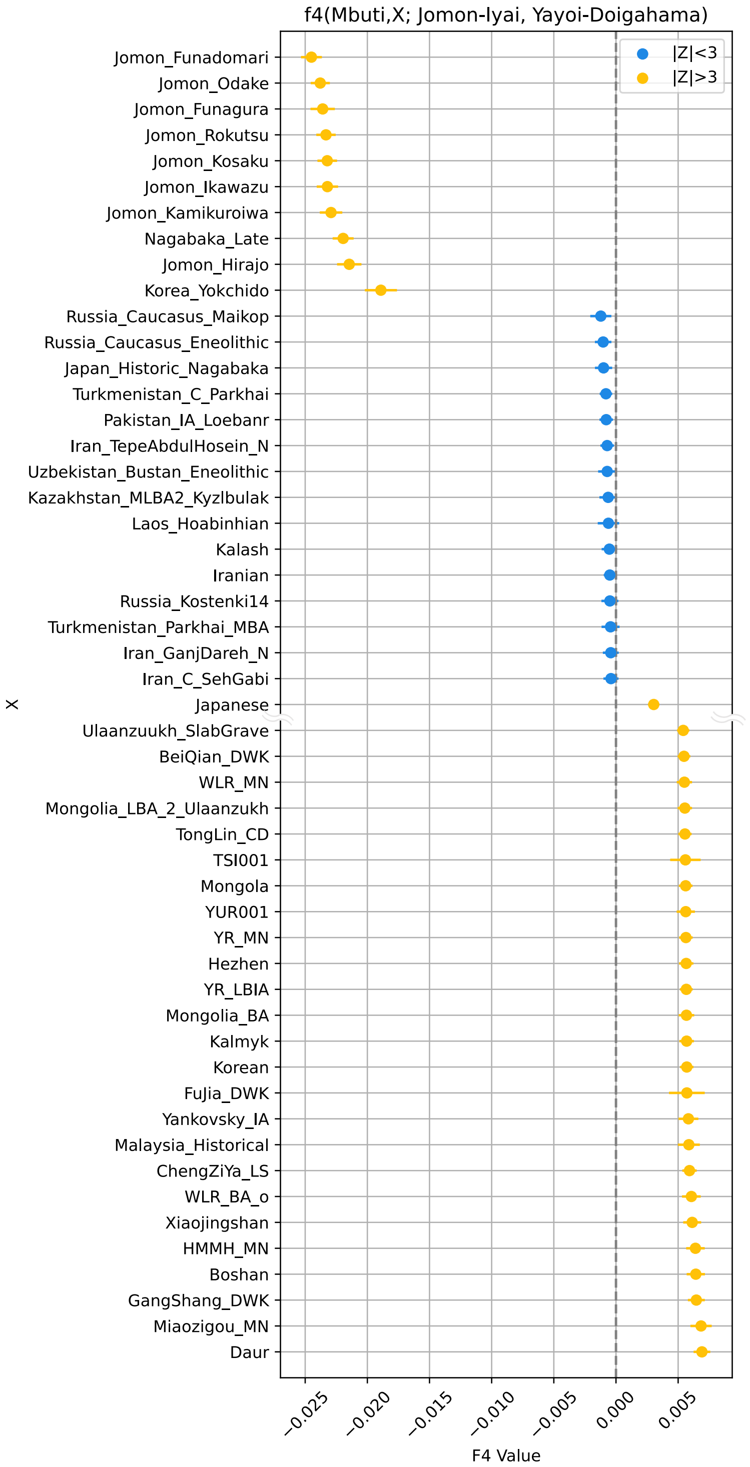

Fig. S16. Shared genetic drift between the Initial Jomon and Middle Yayoi individuals inferred from *f4*-statistics. This figure shows the results of *f4*-statistics of the form *f4*(Mbuti; X, Jomon-Iyai, Yayoi-Doigahama), highlighting population-level affinities with either Initial Jomon (Jomon-Iyai) or Middle Yayoi (Yayoi-Doigahama) individuals. Individuals and populations with the highest absolute f4 values are shown. Error bars represent the standard error. The color scheme highlights bars with |*Z*| > 3 in yellow and |*Z*| < 3 in blue. Negative values indicate stronger genetic affinity with Jomon-Iyai, whereas positive values indicate stronger affinity with Yayoi-Doigahama.

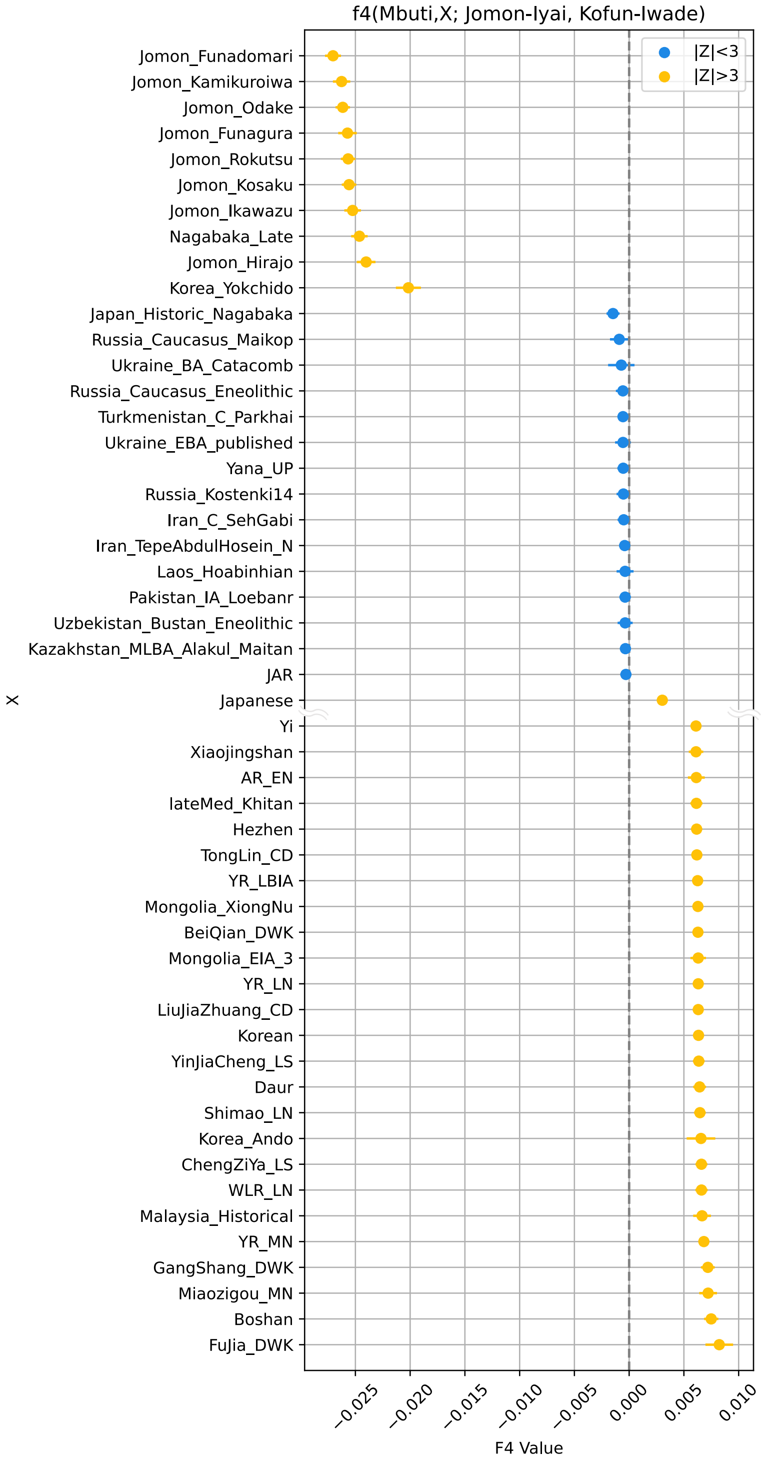

Fig. S17. Results of *f4*-statistics highlighting the shared genetic drift between Initial-Jomon (Jomon-Iyai) and Kofun (Kofun-Iwade). In *f4*(Mbuti; X, Jomon-Iyai, Kofun), the top 50 individuals/populations were selected from those with the highest absolute *f4* values. Error bars indicate the standard deviation; absolute values of *Z* greater than 3 are shown in yellow and values less than 3 in blue. Negative values indicate more shared genetic drift with Jomon-Iyai and positive values with Kofun.

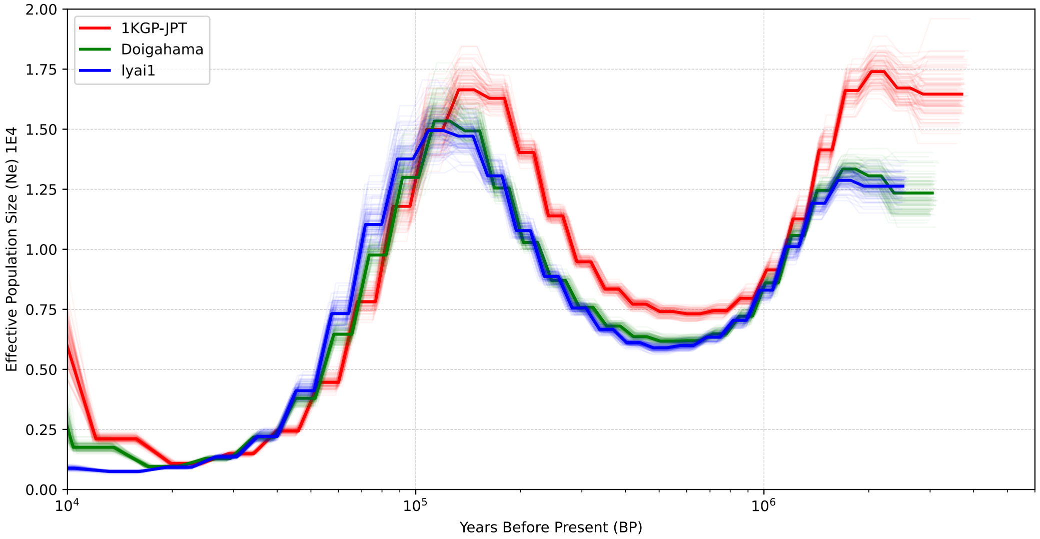

Fig. S18. Demographic inference based on 100 bootstrap replicates using PSMC. This figure shows the results of 100 bootstrap replicates performed with the same parameters as in Fig. 2B. The red line represents the 1KGP-JPT sample, the green line represents the Yayoi Doigahama (DO) individual, and the blue line represents the Jomon Iyai1 (IY1) individual.

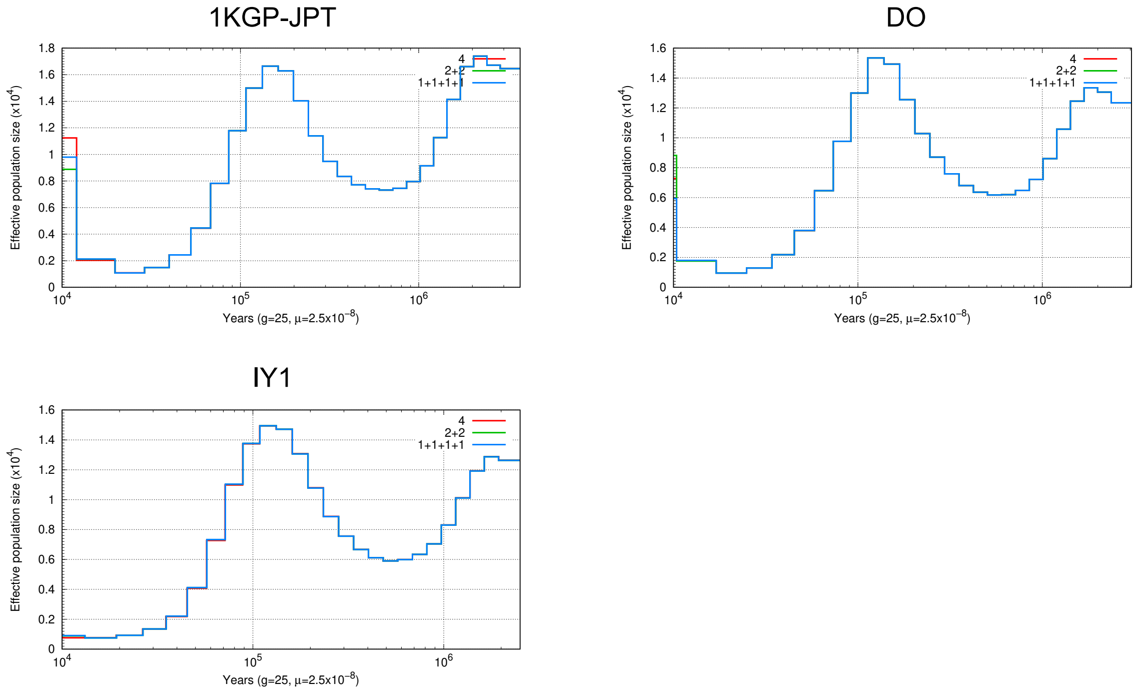

Fig. S19. Demographic inference with PSMC under different first time-slice settings. This figure summarizes the demographic trajectories of 1KGP-JPT, Yayoi Doigahama (DO), and Jomon Iyai1 (IY1) when varying the first time-slice parameter in PSMC to 1+1+1+1 (blue line), 2+2 (green line), and 4 (red line).

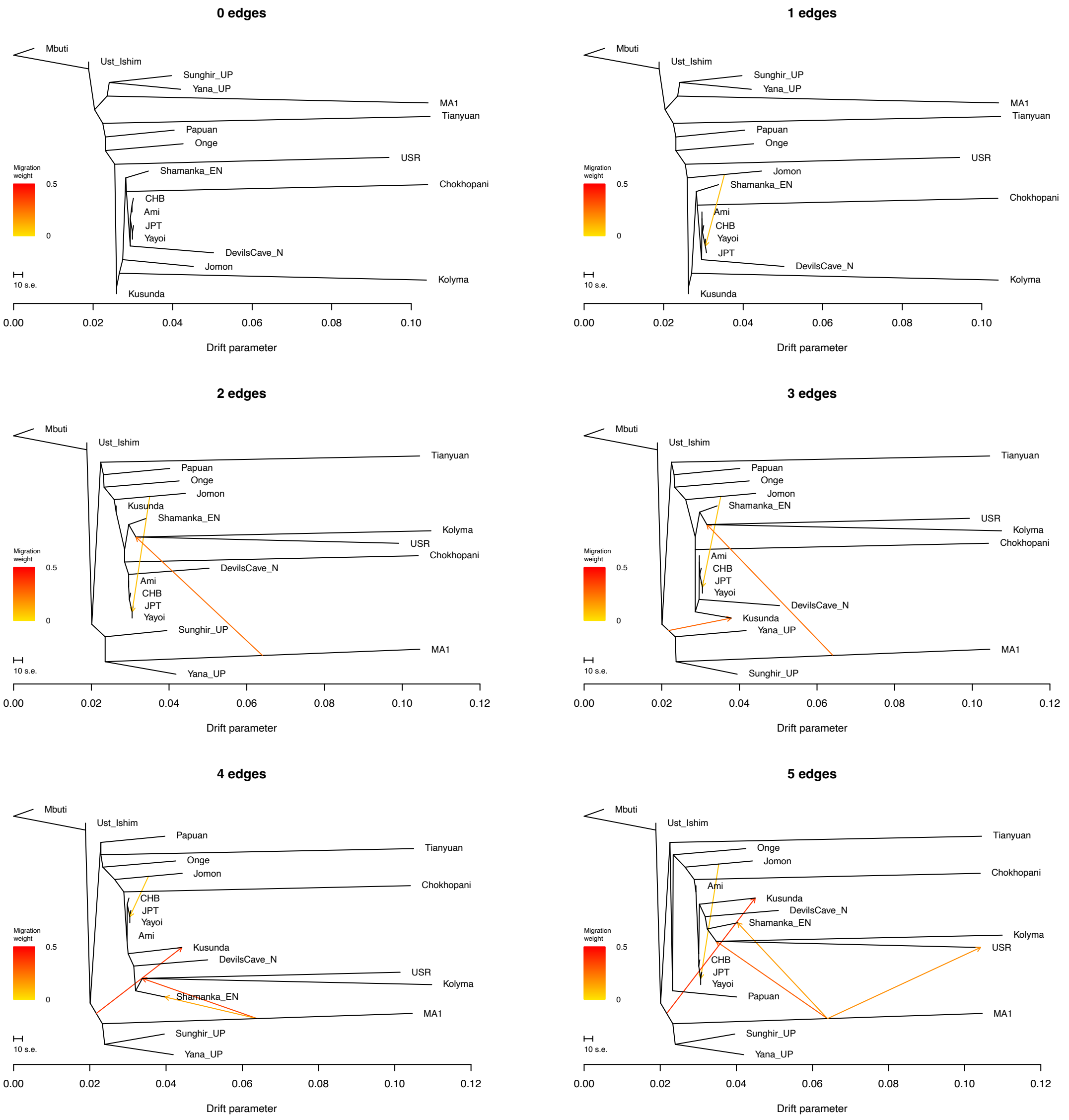

Fig. S20. Maximum likelihood phylogenetic tree of populations under migration models (*m*=0 to 5). The tree shows the phylogenetic relationships between modern and ancient Eurasian populations (ancient: Ust_Ishim, Tianyuan, DevilsCave_N, Shamanka_EN, USR, Kolyma, Chokhopani, Yana_UP, MA1, Sunghir_UP, Jomon (IY1), and Yayoi (DO); modern: Papuan, Onge, Ami, CHB, JPT, and Kusunda; outgroup: Mbuti). The tree represents the results from zero to five as the number of migrations (*m*). The migration weight indicates the fraction derived from the source of ancestral migration.

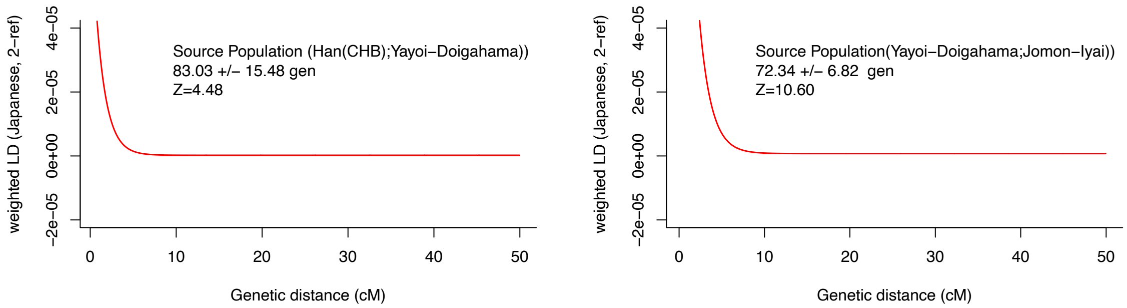

Fig. S21. LD decay curves assuming immigrant population in present-day Japanese population. "Source Population" indicates the reference populations used for modeling LD decay curves in the present-day Japanese population. Han (CHB) from the 1000 Genomes Project, Yayoi-Doigahama (DO), and Jomon-Iyai (IY1) are used as source populations in this analysis.

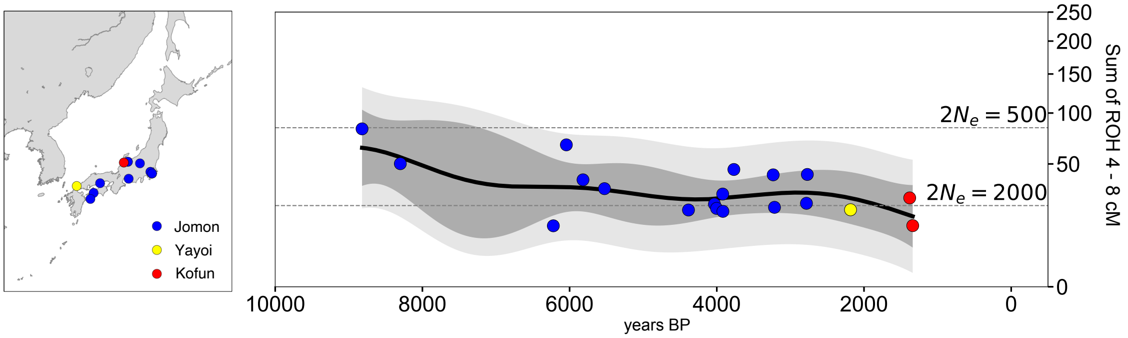

Fig. S22. Time-series variation in runs of homozygosity (ROH) for ancient individuals from the Honshu–Shikoku region in the Japanese Archipelago. The analysis excludes individuals for whom ROH could not be estimated due to insufficient coverage. Scatter points appear color-coded by their cultural ages. The solid black line represents the mean ROH values estimated by a Gaussian Process model. The dark gray area shows the 95% confidence interval of the estimated mean, and the light gray area indicates the confidence intervals for individual estimates. Horizontal dashed lines depict expectations for panmictic population sizes.

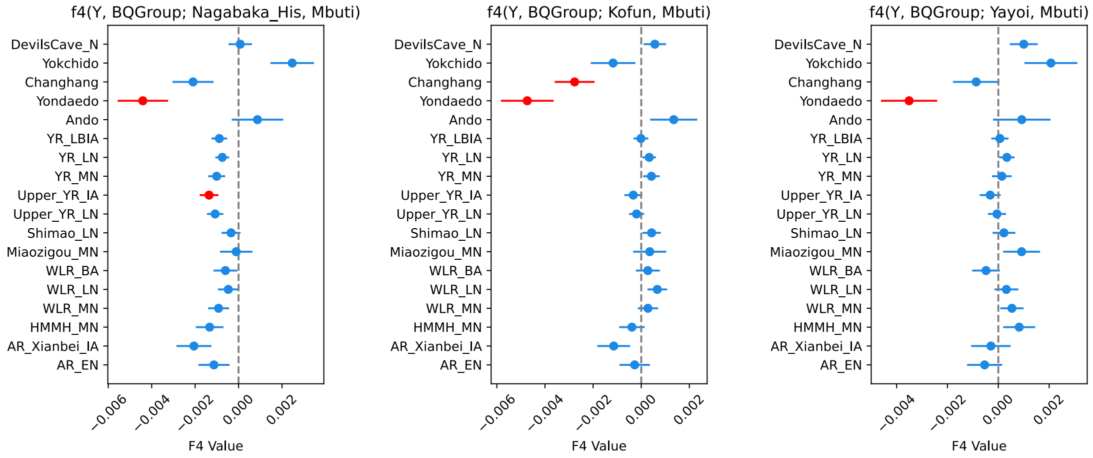

Fig. S23. Differential genetic signals from the BeiQian group in Nagabaka and mainland Japanese populations after the post-Yayoi period**.** Bars are colored red when the absolute *Z*-score is ≥ 3 and blue when it is < 3. Error bars indicate standard errors.

	Population	Phenotype Frequency (%)	Allele Frequency (in_decimals)	Sample Size
	Mexico Sonora Seri		0.5450	34
	Peru Titikaka Lake Uro		0.5000	105
	Peru Titikaka Lake Uros		0.5000	105
	USA Arizona Gila River Amerindian		0.4740	492
	USA Arizona Pima		0.4360	100
	Malaysia Kedah Kensiu	57.0	0.4050	21
	Argentina Gran Chaco Western Toba Pilaga	60.0	0.4000	19
	Finland		0.3440	91
	Mexico Mestizo	56.1	0.3410	41
KOS	Kosovo	52.4	0.3186	124

Figure S24. Screenshot of A*02:01 allele frequencies in modern human populations. The figure displays the top 10 populations with the highest frequencies of the allele.

Population	Phenotype Frequency (%)	Allele Frequency (in_decimals)	Sample Size
Mexico Oaxaca Mixe		0.3490	55
New Zealand Maori with Full Ancestry	52.2	0.2940	46
Mexico Oaxaca Zapotec		0.2610	90
New Zealand Maori with Admixed History	41.0	0.2240	105
Pakistan Kalash		0.2160	69
Mexico Oaxaca Mixtec		0.2160	103
Japan Hokkaido Ainu		0.2000	50
Japan Okinawa Ryukyuan		0.1830	143
Mexico Oaxaca Jamiltepec Mixtec		0.1670	96
Mexico Mixtec		0.1650	97

Figure S25. Screenshot of A*02:06 allele frequencies in modern human populations. The figure displays the top 10 populations with the highest frequencies of the allele.

Population	Phenotype Frequency (%)	Allele Frequency (in_decimals)	Sample Size
China Beijing pop 2		0.4007	826
Japan Hokkaido Ainu		0.2900	50
Sweden Northern Sami		0.1500	154
Mexico Chihuahua Tarahumara		0.1480	44
Sweden Southern Sami		0.1400	130
Mexico Tamaulipas, Ciudad Victoria		0.1304	23
Colombia North Wiwa El Encanto		0.1250	52
China Tibet Region Tibetan		0.1230	158
Netherlands UMCU	23.4	0.1172	64
China Shanxi HIV negative		0.1140	22

Figure S26. Screenshot of B*15:01 allele frequencies in modern human populations. The figure displays the top 10 populations with the highest frequencies of the allele.

Population	Phenotype Frequency (%)	Allele Frequency (in_decimals)	Sample Size
Bulgaria Romani		0.2730	13
India West Bhil		0.1900	50
India Mumbai Maratha		0.1790	91
India Khandesh Region Pawra		0.1700	50
India North pop 2		0.1540	72
South Africa Natal Tamil		0.1430	51
India Andhra Pradesh Golla		0.1300	111
Singapore SGVP. Indian INS		0.1250	86
India Tamil Nadu		0.1116	2492
Iran Baloch		0.1110	100

Figure S27. Screenshot of B*40:06 allele frequencies in modern human populations. The figure displays the top 10 populations with the highest frequencies of the allele.

Population	Phenotype Frequency (%)	Allele Frequency (in_decimals)	Sample Size
Sweden Northern Sami		0.2000	154
USA Alaska Yupik		0.1150	252
Sweden Southern Sami		0.1050	130
USA NMDP Alaska Native or Aleut		0.0890	1376
USA North American Native		0.0860	187
USA Arizona Pima		0.0790	100
USA South Dakota Lakota Sioux		0.0740	302
Belgium	14.3	0.0710	99
USA Arizona Gila River Amerindian		0.0620	492
Finland		0.0610	91

Figure S28. Screenshot of B*27:05 allele frequencies in modern human populations. The figure displays the top 10 populations with the highest frequencies of the allele.

Population	Phenotype Frequency (%)	Allele Frequency (in_decimals)	Sample Size
Colombia North Chimila Amerindians		0.4680	47
Australia Kimberly Aborigine		0.2860	41
Bulgaria Romani		0.2730	13
Papua New Guinea Wosera Abelam		0.2320	131
Australia Yuendumu Aborigine		0.2030	191
Georgia Tibilisi Kurd		0.1550	31
Spain Andalusia Romani		0.1470	99
Australia Groote Eylandt Aborigine		0.1370	75
New Zealand Polynesians with Admixed History	18.5	0.1110	27
USA NMDP South Asian Indian		0.1077	185391

Figure S29. Screenshot of C*15:02 allele frequencies in modern human populations. The figure displays the top 10 populations with the highest frequencies of the allele.

Population	Phenotype Frequency (%)	Allele Frequency (in_decimals)	Sample Size
Mexico Chihuahua Tarahumara		0.3980	44
Brazil Terena	54.0	0.3510	60
Russia Sakhalin Island Nivkhi		0.3210	53
Mexico Chiapas Lacandon Mayans		0.2798	218
China Guizhou Province Miao pop 2		0.2350	85
China Guizhou Province Bouyei		0.2230	109
Papua New Guinea Wosera Abelam		0.2230	131
Mexico Oaxaca Mixe		0.2170	55
USA NMDP Alaska Native or Aleut		0.2060	1376
USA Arizona Gila River Amerindian		0.1950	492

Figure S30. Screenshot of C*03:04 allele frequencies in modern human populations. The figure displays the top 10 populations with the highest frequencies of the allele.

Population	Phenotype Frequency (%)	Allele Frequency (in_decimals)	Sample Size
Cameroon Baka Pygmy		0.2500	10
Russia North Ossetian	40.2	0.2402	127
USA San Francisco Caucasian		0.2130	220
Ireland South	39.2	0.2120	250
Germany Essen	36.2	0.2090	174
Italy South		0.2060	141
Cameroon Sawa		0.1920	13
England North West	35.2	0.1900	298
Thailand Northeast pop 2		0.1840	400
Germany DKMS - Italy minority		0.1826	1159

Figure S31. Screenshot of C*07:01 allele frequencies in modern human populations. The figure displays the top 10 populations with the highest frequencies of the allele.

Population	Phenotype Frequency (%)	Allele Frequency (in_decimals)	Sample Size	IMGT/HLA[1] Database	D
Papua New Guinea East New Britain Tolai		0.5650	48	See	
Papua New Guinea Highland pop2		0.3590	28	See	
Papua New Guinea Highland		0.3590	94	See	
Spain Andalusia Romani		0.3500	99	See	
Sudan Central Region	52.6	0.3100	97	See	
Czech Republic Romani	50.0	0.2928	34	See	
China Shanghai pop 2		0.2880	40	See	
Sudan Mixed	51.0	0.2880	200	See	
Japan pop 2		0.2820	916	See	
South Korea pop 11		0.2790	149	See	

Figure S32. Screenshot of DPB1*02:01 allele frequencies in modern human populations. The figure displays the top 10 populations with the highest frequencies of the allele.

Population	Phenotype Frequency (%)	Allele Frequency (in_decimals)	Sample Size	IMGT/ Data
Papua New Guinea Trobriand Island		0.9760	81	Se
Taiwan Atayal pop 2		0.8000	50	Se
Taiwan Ami pop 2		0.8000	50	Se
Taiwan Rukai pop 2		0.8000	50	Se
Taiwan Bunun pop 2		0.7800	50	Se
Taiwan Saisiat pop 2		0.7600	50	Se
Taiwan Puyuma pop 2		0.7600	50	Se
Papua New Guinea Lowland Roro		0.7080	26	Se
Samoa West		0.7040	22	Se
Taiwan Aborigine pop 2		0.6980	48	Se

Figure S33. Screenshot of DPB1*05:01 allele frequencies in modern human populations. The figure displays the top 10 populations with the highest frequencies of the allele.

Population	Phenotype Frequency (%)	Allele Frequency (in_decimals)	Sample Size
USA Arizona Pima pop2		0.9410	18
USA Arizona Gila River Amerindian		0.8960	492
Colombia North Eastern Plains Sikuani		0.7780	27
Colombia East Amazon Region Nukak		0.7250	20
Russia Siberia Chukotka Peninsula Eskimo		0.6700	80
Russia Siberia Polygus Evenk		0.6600	35
Russia Siberia Eskimo		0.6440	70
USA Alaska Yupik		0.6170	252
Colombia West Waunana		0.6170	30
Brazil Central Plateau Xavante		0.6150	74

Figure S34. Screenshot of DQB1*03:01 allele frequencies in modern human populations. The figure displays the top 10 populations with the highest frequencies of the allele.

Population	Phenotype Frequency (%)	Allele Frequency (in_decimals)	Sample Size
Colombia Sierra Nevada de Santa Marta Ijka pop 2		0.6330	30
Brazil Kaingang		0.5100	235
Australia Kimberly Aborigine		0.4390	41
USA New Mexico Canoncito Navajo		0.4250	42
Colombia Sierra Nevada de Santa Marta Arhuaco		0.4150	107
Mexico Chihuahua Tarahumara		0.3520	44
Peru Titikaka Lake Uro		0.3340	105
Peru Titikaka Lake Uros		0.3330	105
Bolivia La Paz Aymaras		0.3100	87
Bolivia Quechua		0.3043	69

Figure S35. Screenshot of DQB1*04:02 allele frequencies in modern human populations. The figure displays the top 10 populations with the highest frequencies of the allele.

Population	Phenotype Frequency (%)	Allele Frequency (in_decimals)	Sample Size
Colombia Sierra Nevada de Santa Marta Ijka pop 2		0.6170	30
Brazil Kaingang		0.4960	235
USA New Mexico Canoncito Navajo		0.4380	42
Colombia Sierra Nevada de Santa Marta Arhuaco		0.4150	107
Peru Titikaka Lake Uros		0.3240	105
Peru Titikaka Lake Uro		0.3190	105
Mexico Mazahua		0.3100	65
Bolivia La Paz Aymaras		0.3100	87
Mexico Jaltepec Mazahuas		0.3100	50
Mexico Sonora Seri		0.3030	34

Figure S36. Screenshot of DRB1*08:02 allele frequencies in modern human populations. The figure displays the top 10 populations with the highest frequencies of the allele.

Population	Phenotype Frequency (%)	Allele Frequency (in_decimals)	Sample Size
USA Arizona Pima pop2		0.7940	18
USA Arizona Gila River Amerindian		0.7460	492
Colombia East Amazon Region Nukak		0.6500	20
Colombia Waunana NA-DHS_20 (G)	75.0	0.4750	20
Russia Siberia NW of Sakhalin Island Nivkh		0.4200	32
Colombia North Eastern Plains Sikuani		0.4070	27
Colombia Sierra Nevada de Santa Marta Arsario		0.3500	18
Russia Siberia Gvaysugi Udege		0.3500	25
Canada British Columbia Athabaskan		0.3470	62
Canada British Columbia Penutian		0.3460	26

Figure S37. Screenshot of DRB1*14:02 allele frequencies in modern human populations. The figure displays the top 10 populations with the highest frequencies of the allele.

Population	Phenotype Frequency (%)	Allele Frequency (in_decimals)	Sample Size
Russia Siberia Eskimo		0.3610	70
Russia Siberia Chukotka Peninsula Chukchi		0.3000	59
Russia Siberia Chukotka Peninsula Eskimo		0.2800	80
Russia Siberia Chukchi		0.2540	71
USA Alaska Yupik		0.2320	252
Russia Siberia North East Kamchatka Koryak		0.2100	92
Jordan Amman		0.1970	146
Colombia Barranquilla		0.1860	188
Denmark		0.1760	55
Tunisia pop 2		0.1700	111

Figure S38. Screenshot of DRB1*04:01 allele frequencies in modern human populations. The figure displays the top 10 populations with the highest frequencies of the allele.

Population	Phenotype Frequency (%)	Allele Frequency (in_decimals)	Sample Size
Papua New Guinea Kuru		0.3260	46
Spain Pas Valley		0.3210	88
Spain North Cabuerniga		0.3000	95
Papua New Guinea Eastern Highlands Goroka Asaro		0.2980	57
Papua New Guinea Ume		0.2890	90
Papua New Guinea Woigi		0.2880	26
Papua New Guinea Highland		0.2820	94
Malaysia Kedah Baling Kensiu		0.2600	25
Spain North Cantabria		0.2530	83
Papua New Guinea Wonie		0.2160	51

Figure S39. Screenshot of DRB1*15:01 allele frequencies in modern human populations. The figure displays the top 10 populations with the highest frequencies of the allele.

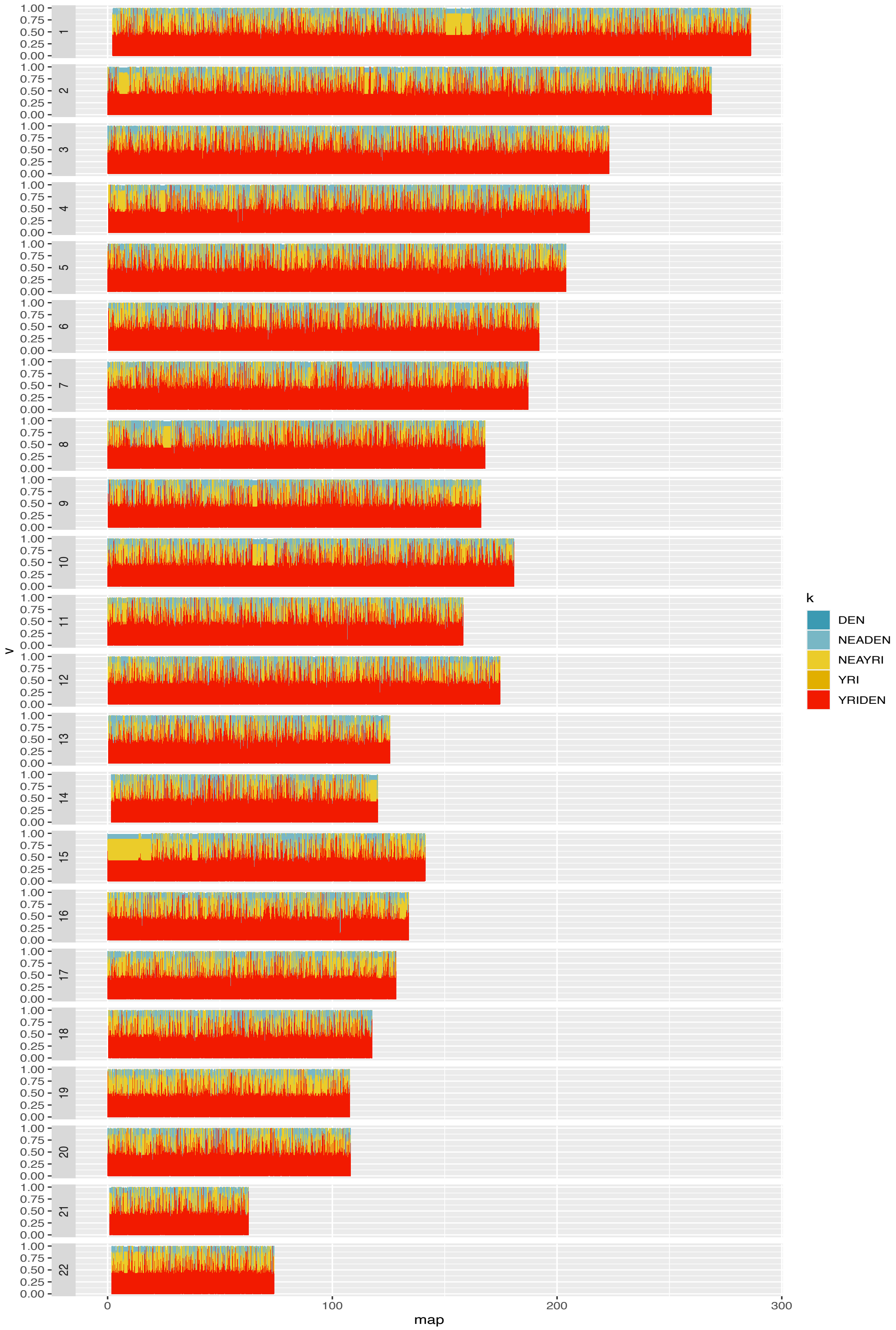

Figure S40. Inference of ancestry fragments in IY1 genome.

Datasets

Dataset S1(separate file). Analysis chart of sample ID, position, and protocol.

Dataset S2(separate file). The list of new and published ancient human genomes in the Japanese archipelago.

Dataset S3(separate file). The list of samples used for population genome analysis in this study.

Dataset S4(separate file). Outgroup *f3* statistics of the form *f3*(Mbuti; DO, X), showing genetic affinities of the Yayoi individual (DO) with East Eurasian populations.

Dataset S5(separate file). Outgroup *f3* statistics of the form *f3*(Mbuti; Kofun-Iwade, X), showing genetic affinities of the Kofun individuals with East Eurasian populations.

Dataset S6(separate file). *f4*-statistics (Mbuti; X, IY1, DO) for shared genetic drift between the Initial Jomon (IY1) and Middle Yayoi (DO) individuals with East Eurasian populations.

Dataset S7(separate file). *f4*-statistics (Mbuti; X, IY1, Kofun-Iwade) for shared genetic drift between the Initial Jomon (IY1) and Kofun (Kofun-Iwade) individuals with East Eurasian populations.

Dataset S8(separate file). Results of qpAdm modeling based on two-way models.

Dataset S9(separate file). Results of *AMY1* copy number estimation.

Dataset S10(separate file). The result of 4-digit alleles at *HLA* locus.

Dataset S11(separate file). The putative archaic segment was identified on IY1 genome.

SI References

1. Y. Taniguchi, K. Asakura, *Iyai Iwakage Iseki: 2014-Nendo Hakkutsu Chousa Houkokusho [Archaeological Research at the Iyai Rock Shelter Site]* (Department of Archaeology, Kokugakuin University, 2017).
2. O. Kondo, M. Yoneda, Y. Taniguchi, A female human skeleton from the Initial Jomon period found in the Iyai rock shelter in mountainous Kanto, Japan. *Anthropol. Sci.* **126**, 151–164 (2018).
3. F. Mizuno et al., Population dynamics in the Japanese Archipelago since the Pleistocene revealed by the complete mitochondrial genome sequences. *Sci. Rep.* **11**, 12018 (2021).
4. C.-C. Wang et al., Genomic insights into the formation of human populations in East Asia. *Nature* **591**, 413–419 (2021).
5. N. P. Cooke et al., Ancient genomics reveals tripartite origins of Japanese populations. *Sci. Adv.* **7**, eabh2419 (2021).
6. H. McColl et al., The prehistoric peopling of Southeast Asia. *Science* **361**, 88–92 (2018).
7. T. Gakuhari et al., Ancient Jomon genome sequence analysis sheds light on migration patterns of early East Asian populations. *Commun. Biol.* **3**, 437 (2020).
8. M. Robbeets et al., Triangulation supports agricultural spread of the Transeurasian languages. *Nature* **599**, 616–621 (2021).
9. H. Kanzawa-Kiriyama et al., Late Jomon male and female genome sequences from the Funadomari site in Hokkaido, Japan. *Anthropol. Sci.* **127**, 83–108 (2019).
10. M. Lipson et al., Ancient genomes document multiple waves of migration in Southeast Asian prehistory. *Science* **361**, 92–95 (2018).

11. V. M. Narasimhan et al., The formation of human populations in South and Central Asia. *Science* **365**, eaat7487 (2019).
12. P. Flegontov et al., Palaeo-Eskimo genetic ancestry and the peopling of Chukotka and North America. *Nature* **570**, 236–240 (2019).
13. M. E. Allentoft et al., Population genomics of Bronze Age Eurasia. *Nature* **522**, 167–172 (2015).
14. M. Meyer et al., A high-coverage genome sequence from an archaic Denisovan individual. *Science* **338**, 222–226 (2012).
15. S. Mallick et al., The Simons Genome Diversity Project: 300 genomes from 142 diverse populations. *Nature* **538**, 201–206 (2016).
16. K. Prüfer et al., The complete genome sequence of a Neanderthal from the Altai Mountains. *Nature* **505**, 43–49 (2014).
17. Q. Fu et al., Genome sequence of a 45,000-year-old modern human from western Siberia. *Nature* **514**, 445–449 (2014).
18. M. Rasmussen et al., The genome of a Late Pleistocene human from a Clovis burial site in western Montana. *Nature* **506**, 225–229 (2014).
19. M. Raghavan et al., Genomic evidence for the Pleistocene and recent population history of Native Americans. *Science* **349**, aab3884 (2015).
20. C. Jeong et al., Long-term genetic stability and a high-altitude East Asian origin for the peoples of the high valleys of the Himalayan arc. *Proc. Natl. Acad. Sci. U.S.A.* **113**, 7485–7490 (2016).
21. F. Broushaki et al., Early Neolithic genomes from the eastern Fertile Crescent. *Science* **353**, 499–503 (2016).
22. I. Mathieson et al., Genome-wide patterns of selection in 230 ancient Eurasians. *Nature* **528**, 499–503 (2015).
23. M. Unterländer et al., Ancestry and demography and descendants of Iron Age nomads of the Eurasian Steppe. *Nat. Commun.* **8**, 14615 (2017).
24. C. Posth et al., Reconstructing the deep population history of Central and South America. *Cell* **175**, 1185–1197.e22 (2018).
25. I. Lazaridis et al., Genomic insights into the origin of farming in the ancient Near East. *Nature* **536**, 419–424 (2016).
26. M. Raghavan et al., Upper Palaeolithic Siberian genome reveals dual ancestry of Native Americans. *Nature* **505**, 87–91 (2013).
27. M. Rasmussen et al., Ancient human genome sequence of an extinct Palaeo-Eskimo. *Nature* **463**, 757–762 (2010).
28. M. Raghavan et al., The genetic prehistory of the New World Arctic. *Science* **345**, 1255832 (2014).
29. A. Seguin-Orlando et al., Genomic structure in Europeans dating back at least 36,200 years. *Science* **346**, 1113–1118 (2014).
30. A.-S. Malaspinas et al., Two ancient human genomes reveal Polynesian ancestry among the indigenous Botocudos of Brazil. *Curr. Biol.* **24**, R1035–R1037 (2014).
31. Q. Fu et al., The genetic history of Ice Age Europe. *Nature* **534**, 200–205 (2016).
32. É. Harney et al., Ancient DNA from the skeletons of Roopkund Lake reveals Mediterranean migrants in India. *Nat. Commun.* **10**, 3670 (2019).
33. M. A. Yang et al., 40,000-year-old individual from Asia provides insight into early population structure in Eurasia. *Curr. Biol.* **27**, 3202–3208 (2017).
34. W. Haak et al., Massive migration from the steppe was a source for Indo-European languages in Europe. *Nature* **522**, 207–211 (2015).
35. 1000 Genomes Project Consortium, A global reference for human genetic variation. *Nature* **526**, 68–74 (2015).
36. M. Lipson et al., Population turnover in Remote Oceania shortly after initial settlement. *Curr. Biol.* **28**, 1157–1165 (2018).
37. M. Mondal et al., Genomic analysis of Andamanese provides insights into ancient human migration into Asia and adaptation. *Nat. Genet.* **48**, 1066–1070 (2016).
38. I. Mathieson et al., The genomic history of southeastern Europe. *Nature* **555**, 197–203 (2018).

39. I. Olalde et al., The Beaker phenomenon and the genomic transformation of northwest Europe. *Nature* **555**, 190–196 (2018).
40. D. J. Kennett et al., Archaeogenomic evidence reveals prehistoric matrilineal dynasty. *Nat. Commun.* **8**, 14115 (2017).
41. M. Sikora et al., Ancient genomes show social and reproductive behavior of early Upper Paleolithic foragers. *Science* **358**, 659–662 (2017).
42. J. V. Moreno-Mayar et al., Terminal Pleistocene Alaskan genome reveals first founding population of Native Americans. *Nature* **553**, 203–207 (2018).
43. A. Mittnik et al., The genetic prehistory of the Baltic Sea region. *Nat. Commun.* **9**, 442 (2018).
44. I. Olalde et al., The genomic history of the Iberian Peninsula over the past 8000 years. *Science* **363**, 1230–1234 (2019).
45. P. de B. Damgaard et al., 137 ancient human genomes from across the Eurasian steppes. *Nature* **557**, 369–374 (2018).
46. K. R. Veeramah et al., Population genomic analysis of elongated skulls reveals extensive female-biased immigration in Early Medieval Bavaria. *Proc. Natl. Acad. Sci. U.S.A.* **115**, 3494–3499 (2018).
47. C. L. Scheib et al., Ancient human parallel lineages within North America contributed to a coastal expansion. *Science* **360**, 1024–1027 (2018).
48. C. de la Fuente et al., Genomic insights into the origin and diversification of late maritime hunter-gatherers from the Chilean Patagonia. *Proc. Natl. Acad. Sci. U.S.A.* **115**, E4006–E4012 (2018).
49. M. Krzewińska et al., Ancient genomes suggest the eastern Pontic-Caspian steppe as the source of western Iron Age nomads. *Sci. Adv.* **4**, eaat4457 (2018).
50. J. Lindo et al., The genetic prehistory of the Andean highlands 7000 years BP though European contact. *Sci. Adv.* **4**, eaau4921 (2018).
51. J. V. Moreno-Mayar et al., Early human dispersals within the Americas. *Science* **362**, eaar6432 (2018).
52. C. Jeong et al., Bronze Age population dynamics and the rise of dairy pastoralism on the eastern Eurasian steppe. *Proc. Natl. Acad. Sci. U.S.A.* **115**, E11248–E11255 (2018).
53. T. C. Lamnidis et al., Ancient Fennoscandian genomes reveal origin and spread of Siberian ancestry in Europe. *Nat. Commun.* **9**, 5018 (2018).
54. C.-C. Wang et al., Ancient human genome-wide data from a 3000-year interval in the Caucasus corresponds with eco-geographic regions. *Nat. Commun.* **10**, 590 (2019).
55. J. Lindo et al., Ancient individuals from the North American Northwest Coast reveal 10,000 years of regional genetic continuity. *Proc. Natl. Acad. Sci. U.S.A.* **114**, 4093–4098 (2017).
56. M. Sikora et al., The population history of northeastern Siberia since the Pleistocene. *Nature* **570**, 182–188 (2019).
57. V. Shinde et al., An ancient Harappan genome lacks ancestry from steppe pastoralists or Iranian farmers. *Cell* **179**, 729–735.e10 (2019).
58. C. Ning et al., Ancient genomes reveal Yamnaya-related ancestry and a potential source of Indo-European speakers in Iron Age Tianshan. *Curr. Biol.* **29**, 2526–2532.e4 (2019).
59. M. Järve et al., Shifts in the genetic landscape of the western Eurasian steppe associated with the beginning and end of the Scythian dominance. *Curr. Biol.* **29**, 2430–2441.e10 (2019).
60. C.-C. Wang et al., The genomic formation of human populations in East Asia. *bioRxiv* [Preprint] (2020).
61. C. Jeong et al., A dynamic 6,000-year genetic history of Eurasia's eastern steppe. *Cell* **183**, 890–904.e29 (2020).
62. J. Liu et al., East Asian gene flow bridged by northern coastal populations over past 6000 years. *Nat. Commun.* **16**, 1322 (2025).
